# Ecologically valid speech collection in behavioral research: The Ghent Semi-spontaneous Speech Paradigm (GSSP)

**DOI:** 10.3758/s13428-023-02300-4

**Published:** 2023-12-13

**Authors:** Jonas Van Der Donckt, Mitchel Kappen, Vic Degraeve, Kris Demuynck, Marie-Anne Vanderhasselt, Sofie Van Hoecke

**Affiliations:** 1 IDLab, Ghent University - imec, Technologiepark Zwijnaarde 122, 9052 Ghent, Zwijnaarde Belgium; 2 Department of Electronics and Information Systems, Ghent University, Ghent, Belgium; 3 Department of Head and Skin, Ghent University, University Hospital Ghent (UZ Ghent), Department of Psychiatry and Medical Psychology, Corneel Heymanslaan 10, 9000 Gent, Belgium; 4 Ghent Experimental Psychiatry (GHEP) Lab, Ghent University, Ghent, Belgium; 5 Department of Experimental Clinical and Health Psychology, Ghent University, Ghent, Belgium

**Keywords:** Acoustics, Speech, Speech collection, Psycholinguistics, Experimental research, Behavioral research, Speech styles, Machine learning

## Abstract

This paper introduces the Ghent Semi-spontaneous Speech Paradigm (GSSP), a new method for collecting unscripted speech data for affective-behavioral research in both experimental and real-world settings through the description of peer-rated pictures with a consistent affective load. The GSSP was designed to meet five criteria: (1) allow flexible speech recording durations, (2) provide a straightforward and non-interfering task, (3) allow for experimental control, (4) favor spontaneous speech for its prosodic richness, and (5) require minimal human interference to enable scalability. The validity of the GSSP was evaluated through an online task, in which this paradigm was implemented alongside a fixed-text read-aloud task. The results indicate that participants were able to describe images with an adequate duration, and acoustic analysis demonstrated a trend for most features in line with the targeted speech styles (i.e., unscripted spontaneous speech versus scripted read-aloud speech). A speech style classification model using acoustic features achieved a balanced accuracy of 83% on within-dataset validation, indicating separability between the GSSP and read-aloud speech task. Furthermore, when validating this model on an external dataset that contains interview and read-aloud speech, a balanced accuracy score of 70% is obtained, indicating an acoustic correspondence between the GSSP speech and spontaneous interviewee speech. The GSSP is of special interest for behavioral and speech researchers looking to capture spontaneous speech, both in longitudinal ambulatory behavioral studies and laboratory studies. To facilitate future research on speech styles, acoustics, and affective states, the task implementation code, the collected dataset, and analysis notebooks are available.

**Supplementary Information:**

The online version contains supplementary material available at 10.3758/s13428-023-02300-4.

## Introduction

Over the past few decades, the human voice and speech have been increasingly studied in relation to, amongst others, psychiatric disorders (e.g., depression, schizophrenia), and current psychological (e.g., stress) or physiological (e.g., sleepiness) states (Fagherazzi et al., 2021; Van Puyvelde et al., 2018). To date, the primary form of speech data used in affective-behavioral research in an experimental setting remains scripted read-aloud speech gathered in highly controlled laboratory environments (Van Puyvelde et al., 2018; Wagner et al., 2015). Scripted lab speech more conveniently allows for systematic experimental control, thus limiting the implicit inclusion of unwanted latent variables. As a result, a smaller sample size is sufficient to capture all degrees of freedom compared to unscripted speech gathered in less controlled environments (Xu, 2010). However, acoustic properties found in one speech style can be style-specific, which limits the explanatory power of the speech data to other speech styles (Ernestus et al., 2015; Wagner et al., 2015). Therefore, a promising research direction is to investigate the influence of speech collection paradigms on both production and perception (Wagner et al., 2015). Such research should also examine the extent to which (affective) findings can be generalized across various speech registers. Furthermore, the scalability of speech elicitation methods should be considered, given that the long-term objective of affective sensing experiments is to facilitate wide-spread, real-world affect monitoring at a near continuous scale (Kappen et al., 2023; Slavich et al., 2019). To this end, it is necessary to investigate speech collection approaches that can be used, next to lab settings, in real-life environments, which facilitate repeated measures, but still allow for sufficient experimental control. As such, this would allow for translation between results concluded from lab collected speech and real-world setting collected speech, as long as the same collection approach has been used.

Prior work has indicated that vocal responses to affective loads may be as individual and unique as the voice itself, requiring more isolated studies that control for inter-individual differences (Giddens et al., 2013; Van Puyvelde et al., 2018). In order to address this issue, within-subject designs have been proposed, which allow for the collection of both baseline and affective data (Giddens et al., 2010, 2013; Kappen  et al., 2022a, 2022b; Van Puyvelde et al., 2018). However, in these works, the acoustic analysis was conducted on read-aloud speech with a fixed text, which limits the generalizability of conclusions to the more naturalistic and spontaneous speech encountered in real-life settings. It has been demonstrated that affective states can influence decisions, working memory, and information retrieval (Mikels & Reuter-Lorenz, 2019; Weerda et al., 2010). Therefore, unscripted speech, which requires larger planning units such as sentences, clauses, and temporal structure, can lead to changes in wording, grammar, and timing of speech under these affective states (Fromkin, 1973; Paulmann et al., 2016; Slavich et al., 2019). These prosodic markers are less pronounced in scripted speech, as fewer planning units are needed (Barik, 1977; Xu, 2010). Baird et al. (2019) tackled the within-participant challenge by developing data-driven models which predict cortisol concentration as a target based on acoustic features. Their spontaneous speech samples were acquired using the Trier Social Stress Tests (TSST; Kirschbaum et al., 1993). In more recent work, Baird et al. (2021) assessed the generalizability of spontaneous speech correlates for stress via cortisol, heart rate, and respiration, by using three TSST corpora. The results show an increasing trend towards generalization and explanation power. However, these results are still limited, as the TSST only produces stressed speech under psychosocial load (i.e., during the interview), without consensus on the collection of baseline speech.

Spontaneous speech rarely allows for controlling the factors that contribute to the phenomena of interest (Xu, 2010). To address this, more controlled variants of unscripted speech paradigms are employed, such as guided interviews and picture description tasks. For example, language disturbances, at both the acoustic-prosodic and content level, have been shown to be promising markers for psychiatric diseases such as schizophrenia-spectrum disorders (de Boer et al., 2020). As a result, schizophrenia researchers have employed guided interview protocols as a means of acquiring unscripted speech (Voppel et al., 2021). Recent work in this area has proposed more continuous disorder follow-up, for which such labor-intensive interviews may not be an ideal match (de Boer et al., 2021). Besides guided interviews, researchers have used picture description tasks (i.e., providing an image stimulus to a participant with the instruction to describe the image content out loud) in the field of neurology, such as aphasia and Alzheimer’s (Goodglass et al., 2001; Mueller et al., 2018). Semi-spontaneous picture description paradigms are here preferred over spontaneous speech, as the controlled and monological types of content are easier to obtain and analyze in clinical practice (Lind et al., 2009; Tucker & Mukai, 2023). Furthermore, by letting participants describe stimuli with consistent emotional loads, repeated measures are possible with little change in affect (Helton & Russell, 2011; Kern et al., 2005).

Given the above observations, in addition to other insights gleamed from previous studies and researchers, as well as our laboratory’s own direct experiences, we established a requirement list for a speech collection task that would be useful for both experimental research and real-world applicability (Kappen et al., 2023; Kappen et al., 2022b; Slavich et al., 2019; Wagner et al., 2015; Xu, 2010). The task should (1) allow for flexible speech recording durations, ensuring that it can easily be incorporated into existing paradigms. For example, enabling the inclusion of a task at multiple (time-constrained) moments within an experiment allows for within-participant analysis. Additionally, the task should be (2) straightforward and non-interfering, ensuring that the resulting speech is not affected by the cognitive-emotional load of the collection method itself, but only by prior effects induced by the experimental paradigm. The method should be (3) controllable, as experimental control reduces the large number of samples that would be needed elsewhere to marginalize out latent factors (Xu, 2010). Furthermore, the method should (4) stimulate participants towards spontaneous speech, as the richness in prosody, semantics, and content has already been proven to be useful to derive markers in affective and cognitive research (Christodoulides, 2016). Unscripted speech should also be more generalizable to everyday speech, enabling the translation of results to real-world settings and applications. Finally, the speech elicitation method should be (5) scalable, by requiring minimal human interference during recording to allow for usability in both longitudinal ambulatory studies with repeated measures and studies at scale.

This paper aims to make a significant step towards the application of lab results in a real-world setting by introducing the *Ghent Semi-spontaneous Speech Paradigm* (*GSSP*), a controllable and ecologically valid picture description paradigm that complies with the above requirements. By having participants describe an image depicting a neutral social setting that is not complex and that they have not seen before, there will be no cognitive interference of active recall. Whereas speech analysis for (psychosocial) stress and other psychological states is increasingly gaining traction, we propose these stimuli to be congruent with psychosocial (stress) paradigms. That is, offering stimuli that would minimally interfere with elicited psychophysiological states of the experimental paradigm in order to (1) not risk the disruption of observed effects in other constructs (e.g., physiological reactions, rumination, etc.) due to mind wandering and (2) have the collected speech closely resemble the active mental state experienced by participants due to the experimental paradigm. In accordance with the terminology of Tucker & Mukai (2023), the GSSP produces unscripted semi-spontaneous speech, given that there is a control on the context and content.

The selected images are empirically sampled from the PiSCES (Teh et al., 2018) and Radboud (Langner et al., 2010) datasets, based on peer-rated neutral content. In order to minimize additional cognitive task load and biases, we used proper habituation instructions and images with a consistent neutral emotional load. To the best of our knowledge, this is the first work proposing a picture description task for applied/real-world acoustic analysis of affective-behavioral states.

To summarize, the contributions of this paper are threefold:

- We propose the Ghent Semi-spontaneous Speech Paradigm (GSSP), a novel speech collection paradigm using a picture description task for affective-behavioral research. The GSSP enables relatively low-effort, semi-controlled recording of unscripted speech data in both experimental and longitudinal real-life settings.
- To assess the validity of the GSSP regarding speech style, utterance duration, and image subset consistency, a study was performed using a web application. The analysis of the web application data indicated that participants were able to describe the images with sufficient duration to extract core speech features (i.e., longer than 15 seconds), and acoustic analysis suggested that the acoustic properties of the GSSP correspond to those of spontaneous speech.
- In order to facilitate the reproducibility of the research outcomes, the materials utilized in the study have been made openly accessible under a research-friendly license. The analysis scripts and web-app code are available on GitHub^1^, while the dataset and instruction videos can be accessed through Kaggle datasets^2^.

Methods: Web app for paradigm validation

In order to evaluate three key factors pertaining to the GSSP, namely (1) the participant's ability to engage in prolonged discourse, (2) the acoustical similarity between the gathered GSSP speech and spontaneous speech, and (3) the consistency of the initially selected image subset, a web application was developed which incorporates the GSSP among a standardized read-aloud task. The following sections describe the web app design and the GSSP procedure, followed by a specification of the participant selection procedure and the speech data processing.

## Web app and procedure

**Fig. 1:**
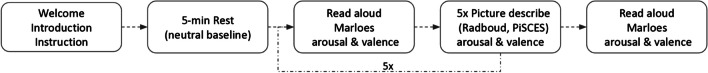
Flowchart of the web application experiment. *Note*. This results in 7 Marloes, 15 Radboud, and 15 PiSCES utterances per participant

**Fig. 2:**
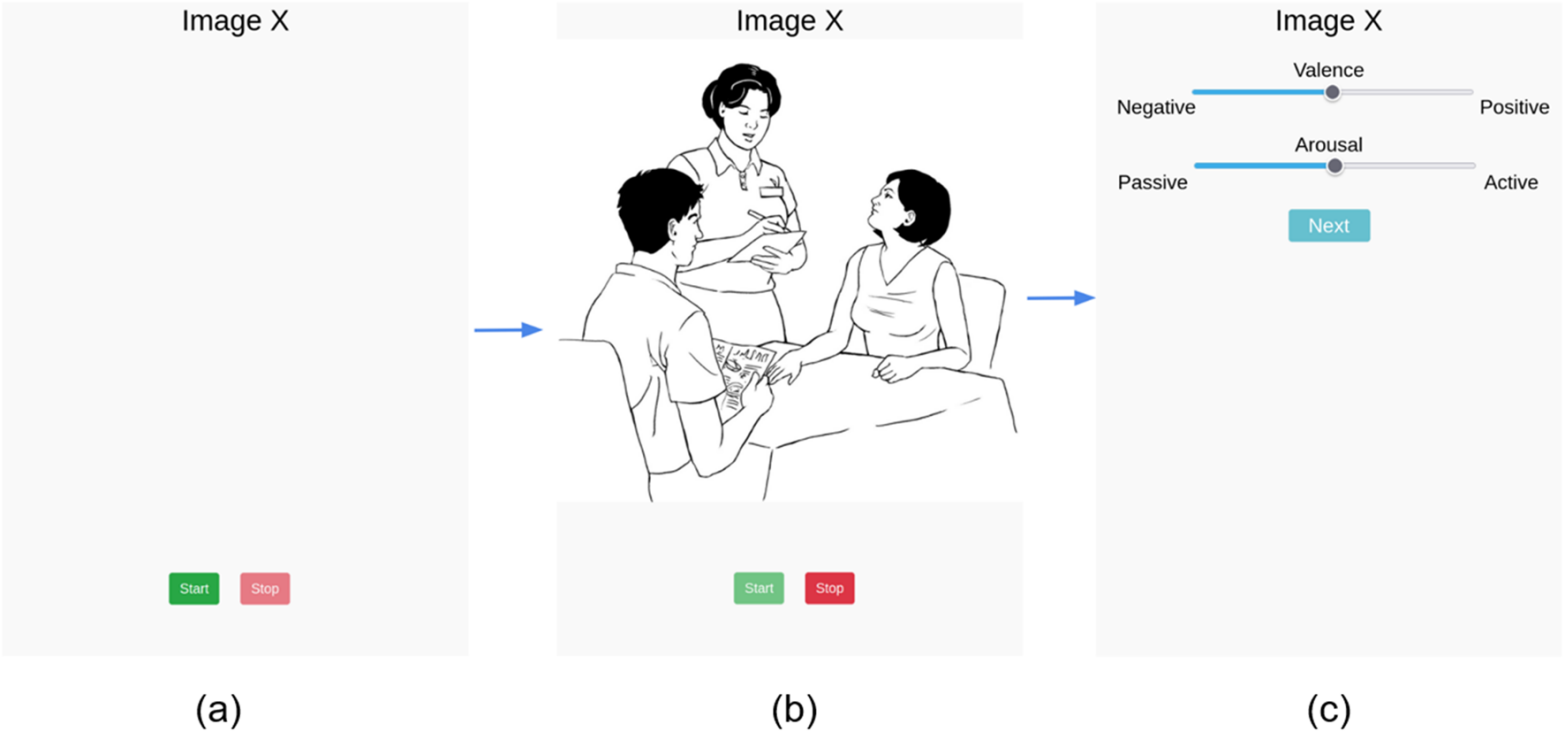
Trial flow chart of the web app speech collection task, with the pages translated to English. First, an empty page (**a**) is displayed with an enabled start button and a disabled stop button. When the participant clicks the start button, (**b**) the audio recording begins, the stop button will be enabled. The stimulus in the form of an image (or text for the read-aloud task) is being presented. After the participant completes the stimulus speech collection task, he/she or they click on the stop button, triggering the redirection to (**c**), where the participant reports their experienced arousal and valence values

**Fig. 3:**
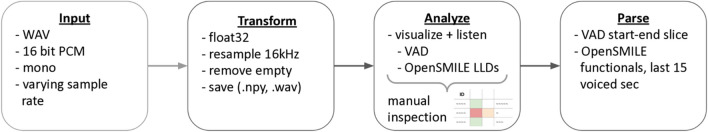
Audio data processing flowchart

**Fig. 4:**
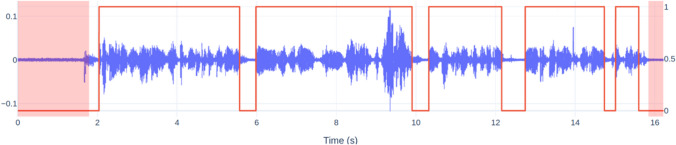
VAD slicing with a 0.25 s margin for the first and last voiced segment. *Note.* The first voiced regions occur approximately 2 seconds after the participant pressed the “start” button. The slicing ensures that each participant's first/last voiced segment start/end at the same time, allowing to make fair comparisons on fixed-duration excerpts relative from the VAD-slice beginning or end

**Fig. 5:**
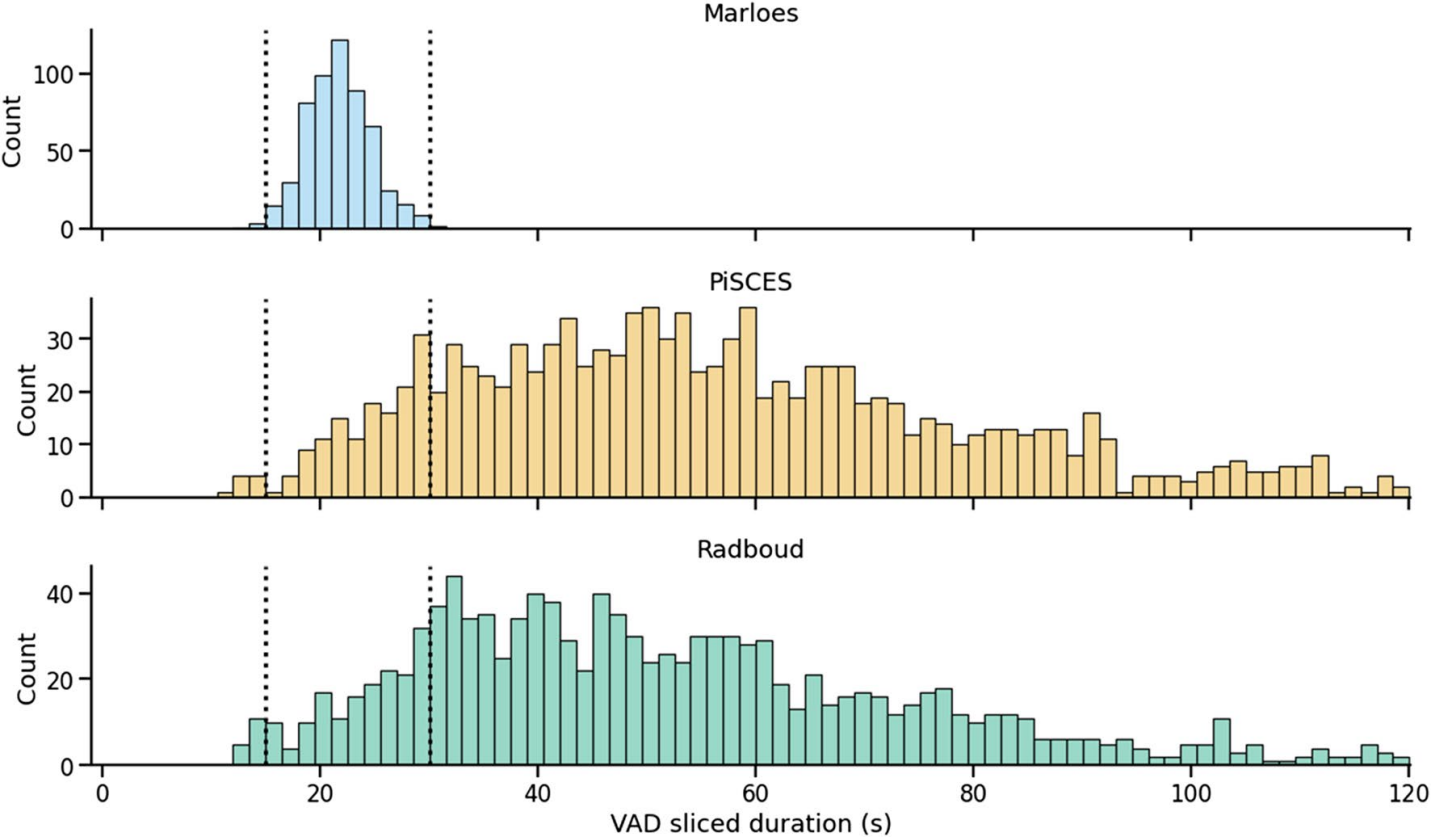
Distribution plot of the VAD-sliced utterance durations. The vertical dashed lines on the left indicate the voiced duration threshold (15 seconds) and the lines on the right represent the instructed image description duration (30 seconds)

The web application was developed in Python using the Flask framework (Grinberg, 2018). Screenshots and implementation details are found in Supplemental Material S1 and on GitHub^3^. As depicted in Fig. 1, the experiment was divided into five blocks, with the first block consisting of three consecutive web pages. The first page, labeled “Welcome” (S1.1 Figure 1), provided a general overview of the study’s purpose, i.e., validating the usability of an image set for experimental speech research. The second page, labeled "Introduction” (S1.2 Figure 2), was used to acquire demographics (i.e., age, sex, recording material, highest obtained degree) together with the approval of the informed consent. The introduction page also provided an overview of the general guidelines for the task. In particular, it emphasized the importance of performing the task on a computer in a quiet and distraction-free environment. The complete list of (translated) guidelines can be found in S1.2. The third page, labeled “Task Instruction” (S1.3 Figures 3, 4 and 5), provided detailed instructions for the components of this study, i.e., a 5-minute resting block (S1.4) to establish a neutral baseline state, followed by the speech collection tasks through scripted read speech (i.e., “Marloes”) and the GSSP. The task instruction page also provided three videos. The first video demonstrated the procedure for the reading task, in which the Marloes text is read-out loud with a normal reading voice. The subsequent two videos illustrated the GSSP picture description process, utilizing representative images from both the PiSCES and Radboud datasets. It is important to note that the chosen images from these datasets were not utilized as stimuli in the study. In addition, the instruction page presented the read-out-loud (“Marloes”) text and participants were instructed to read the text out loud. This reading exercise, together with the demonstration videos, aimed to reduce novelty effects for both the GSSP and reading task (Davidson & Smith, 1991; Weierich et al., 2010; Zuckerman, 1990). The study asked participants to provide a description of each image for a minimum of 30 seconds, but no explicit instruction was given to adhere to this duration, nor was the length of the speech recording indicated to the subjects. Finally, as a speech quality control procedure, participants had to record and playback a speech sample, and were only permitted to proceed to the resting block after this microphone assessment was conducted.

The resting block consisted of a blank page featuring the text: “*Close your eyes and try to focus on your breathing. You will hear a sound when the resting block is over*” (translated). This step aimed to bring the participants to a neutral baseline state and is in alignment with Kappen et al. (2022a) and Kappen, Van Der Donckt, et al. (2022b).

Afterward, participants performed six iterations through the third and fourth block, resulting in six read-aloud segments and 30 GSSP speech samples. Finally, as shown by Fig. 1, a read-aloud sample was acquired in the fifth block, upon which the study was completed.

### Read-out-loud text “Marloes”

To acquire scripted speech fragments, participants were instructed to read aloud a standardized text of five sentences. The text, commonly known as the “Marloes” text, is widely used in Dutch speech therapy due to its phonetic balance (Van de Weijer & Slis, 1991; full text provided in S1.5). As depicted in the speech collection flow of Fig. 2, the “Marloes” text only became visible after the participant initiated the task by clicking the start button, which should limit the variability in preparation time. Once the segment has been read out loud, participants could proceed to a new page by clicking the stop button. On this page, two sliders were presented, which participants adjusted to indicate their level of arousal and valence experienced during the speech task (Fig. 2).

GSSP picture description speech

The unscripted speech fragments were collected in accordance with the read-aloud task. In order to limit the variability of image description preparation time, all stimuli were presented to the participants at the beginning of the recording upon clicking the start button. This approach ensured a degree of uniformity among participants. The order of the presented images was randomized, alternating between pictures from the PiSCES and Radboud databases. The first image shown was drawn from the PiSCES subset, followed by an image from the Radboud set, and so on. Each cycle consisted of a total of five pictures, resulting in a total of 15 images from both the PiSCES and Radboud databases (as shown in Fig. 1). To ensure optimal audio quality, speech data was stored within the participant’s browser session using the Recorderjs JavaScript tool (Matt, 2016). After utterance completion, the audio data was converted into a 16 bit PCM mono WAV file and sent to a secure server, along with the experienced arousal and valence score.

The PiSCES database is a collection of 203 black-and-white line drawings of individuals in social settings (Teh et al., 2018). These stimuli were evaluated based on emotional valence, intensity, and social engagement. To control for emotional responses, a subset of 15 images with neutral valence ratings and high social engagement scores were selected from this database for use in the study. The images are illustrated in Supplemental S1.6. Figure 7.

Similarly, the Radboud Faces Database provides a set of stimuli including both adult and children's faces that have been parametrically varied with respect to displayed expressions, gaze direction, and head orientation (Langner et al., 2010). These stimuli were evaluated based on the facial expression, valence, and attractiveness. The GSSP utilizes a subset of the neutral expression, front-facing adult images (seven male, eight female), which were selected based on their proximity of average valence scores to neutrality in order to minimize the potential for inducing emotional responses in respondents. The image subset used is depicted in Supplemental S1.6. Figure 8.

### Drinking pause

To mitigate vocal fatigue, participants were instructed to take a sip of water after every nine utterances (Welham & Maclagan, 2003).

## Participants

The data were collected in two waves. First, the research groups’ networks were leveraged by distributing the study via social network sites. Second, the Prolific platform (Palan & Schitter, 2018) was utilized to gain an adequate number of participants. This resulted in a convenience sample of 89 participants (45 female, 43 male, 1 other) with an average age of 27.54 years (*SD* = 6.63). The study included only Dutch-speaking participants residing in Belgium or the Netherlands whose native language was Dutch. On average, participants required 1 hour to complete the study.

Data processing

The audio data parsing and analysis was carried out in Python 3.8.13, and statistical analyses of the valence-arousal scores were performed using R4.1.1. For detailed version information of the utilized libraries, we refer to the GitHub repository^4^.

### Audio data processing

The audio data processing workflow is depicted in Fig. 3. The first step is to acquire the input samples (Input step), which are then converted (Transform step) to 16 kHz mono audio with 32-bit float precision. Due to technical issues, some recordings were not saved properly, resulting in empty audio-files that are excluded from further analysis during the Transform step. The non-empty transformed outputs are then saved for further processing in the Analyze and Parse steps. Following the Transformation step, a participant-level manual inspection is carried out to assess the audio data quality (Analyze step). The inspection process involves utilizing customized visualizations, as illustrated in Fig. 4 and S2 Figure 9, to assist in the analysis process. The outcome of this analysis is a manual inspection sheet, which is used to exclude participants with inadequate audio quality. Lastly, a parsing step is performed on the transformed audio for participants whose audio quality was deemed sufficient. This parsing step employs a voice activity detection (VAD) model (*Speechbrain/Vad-Crdnn-Libriparty · Hugging Face*, n.d.) from the SpeechBrain toolkit (Ravanelli et al., 2021) to detect speech segments. The outer bounds of the first and last speech segments are padded with a margin of 0.25 seconds before slicing. The red shaded regions in Fig. 4 illustrates the regions that are omitted. As such, each VAD-sliced segment consists of speech data that starts and ends at the same relative time. This approach allows us to make fair comparisons between fixed duration excerpts (relative from VAD-slice beginning or end). Supplemental S2 further details the visualizations that are utilized during the “Analyze” step.

### Acoustic speech parameter extraction

The final stage of the parsing step entails the extraction of speech parameters. To control for the effects of file duration on acoustic parameters and repetitive start sentences in the picture description tasks (e.g., “*I see a black and white cartoon*” for the PiSCES database), only the last 15 voiced seconds, as determined by the VAD-slice, were used for both parameter extraction techniques listed below. Therefore, only excerpts with a VAD-slice duration of at least 15 seconds were included, resulting in 2901 samples from 82 participants (554 Marloes, 1184 PiSCES, 1163 Radboud). The number of removed recordings per participant is portrayed in Supplemental S3, Figure 11.

The extraction of speech parameters was conducted using the openSMILE 3.0.1 Python API (Eyben et al., 2010) and the GeMAPSv01b functional configuration (Eyben et al., 2016). The selection of the GeMAPSv01b configuration was in line with previous research (Baird et al., 2019, 2021; Jati et al., 2018; Kappen et al., 2022a, 2022b). Moreover, Triantafyllopoulos et al. (2019) observed that the eGeMAPS, which is a superset of the GeMAPS, is relatively robust in noisy conditions. A comprehensive explanation of the utilized openSMILE feature subset can be found in Supplementals S4. During the manual inspection phase of the Analyze step (as illustrated in Fig. 2), differences in the values of openSMILE low-level descriptors (LLDs) were observed when the original 44.1 kHz data were resampled to 16 kHz. Further examination of openSMILE’s sampling-rate inconsistencies is available in Supplemental S5. This examination led to superposing a small (Gaussian-sampled) noise of −30 dB to the resampled audio, which empirically improved the voiced boundary detection.

In addition to the acoustic parameter investigation, visual speech style analysis was performed via deep learning embeddings, generated using the ECAPA-TDNN architecture (Desplanques et al., 2020). These embeddings were projected into a two-dimensional space using t-SNE (Van der Maaten & Hinton, 2008). Further implementation details regarding the GeMAPSv01b and ECAPA extraction procedures can be found in the feature extraction and *ECAPA-TDNN* notebooks, respectively^5^.

Finally, to evaluate the binary separability of speech styles in a data-driven manner, the openSMILE features and ECAPA-TDNN embeddings were also fed to a machine learning model. Specifically, logistic regression, a linear classification model, was used to assess this separability. The Scikit-learn Python toolkit by Pedregosa et al. (2011) was used for this purpose.

### External dataset “Corpus Gesproken Nederlands”

To validate the generalizability of the data-driven speech style assessment, an external dataset was utilized. Specifically, a subset of the *Corpus Gesproken Nederlands* (CGN), i.e., the Corpus of Spoken Dutch, was leveraged (Oostdijk, 2000). CGN includes recordings of both Flemish and Netherlands Dutch, which are categorized into various components based on speech style and context settings. These components range from spontaneous conversations and news broadcasts, to sport commentaries, sermons, and read-aloud texts. The corpus data is stored as 16-bit PCM 16 kHz WAV files, and each recording is orthographically transcribed and diarized.

Two components were chosen from the CGN dataset to serve as our unscripted and scripted speech styles. Component A, “face-to-face conversations”, was deemed unsuitable for the unscripted speech style due to the presence of frequent interruptions and crosstalk in the recordings. Component B, “interviews with Dutch teachers”, was used as unscripted speech style data because the data has low emotional load and interviewee’s utterances have few interruptions and often meet the 15-second duration criterion. Finally, Component O, “read-aloud texts”, served as scripted speech style data in our validation. In accordance with the acoustic parameter extraction performed on the web app data, excerpts of the last 15 seconds (with a margin of 2 seconds) were taken from single speaker segments that met the duration requirement and the openSMILE GeMAPS v01b configuration was applied. This resulted in a validation dataset of 3357 segments (1643 scripted read speech [comp. O] and 1714 spontaneous speech [comp. B]). The utterances from the validation dataset originate from 301 speakers, consisting of 163 female, 138 male, with an average age 43.12 years (SD = 14.33). Notably, there is no speaker overlap between the two components. Component specific characteristics are further detailed by Table 1.

**Table 1:** Characteristics of selected segments from comp. B and comp. O

	Comp. B	Comp. O
Speech style	unscripted (interviewee)	scripted (read-aloud)
Number of segments	1714	1634
Number of speakers	114	187
Speaker sex of each segment: F/M	962/752	884/759
Age: segment based mean (SD)	41 (11)	45 (16)

## Results

This section presents the results of the web app data analysis. In the first subsection, we focus on the affective consistency of the GSSP stimuli and present the arousal and valence scores. Next, the speech style of GSSP is analyzed using renowned acoustic features in relation to existing literature on speech styles. The jitter and shimmer features trended differently from prior research, prompting a subsection containing a detailed exploration of this inconsistency. The GSSP speech style is further evaluated using data-driven methods, including an ECAPA-TDDN t-SNE projection for analysis and generalizability of the GSSP towards unscripted speech styles beyond the web app dataset.

## Arousal and valence scores

As described in the methods section, the PiSCES and Radboud database stimuli were selected by choosing the closest to the middle of the valence scale in its respective validation studies, whilst accounting for potential thematic difficulties that could elicit certain emotional responses in subgroups of people. In doing so, we have compiled a picture subset that could be considered emotionally neutral and therefore appropriate in affective research. Additionally, we have conducted a series of statistical and descriptive approaches to also validate the appropriateness of our picture subset. These tests can be found on the analysis repository^6^, as they are not key findings in this manuscript, yet are of importance to assess the rigidity and validity of the results presented here.

In longitudinal studies, we recommend randomizing stimuli. Although we noticed variability in our stimuli, it remains sufficiently limited to warrant randomization. Researchers can opt to use all 15 images or select a subset suitable for their test frequencies, referencing the supplemental data provided for each image. Some study designs might prioritize consistent valence, while others might focus on arousal.

## Speech feature analysis

### Speech duration

The web app guidelines, as outlined in Supplemental S1.2., instructed the participants to discuss each image for a minimum of 30 seconds. The speech task histograms in Fig. 5 demonstrate that 88% of the GSSP utterances adhered to this duration requirement. It is worth noting that the 30-second threshold exceeds the longest voiced duration observed in the "Marloes" task. Consequently, this suggests that, for at least 88% of the GSSP samples, a greater amount of voiced data is available compared to what would be obtained by utilizing the Marloes task.

### openSMILE acoustics

**Fig. 6:**
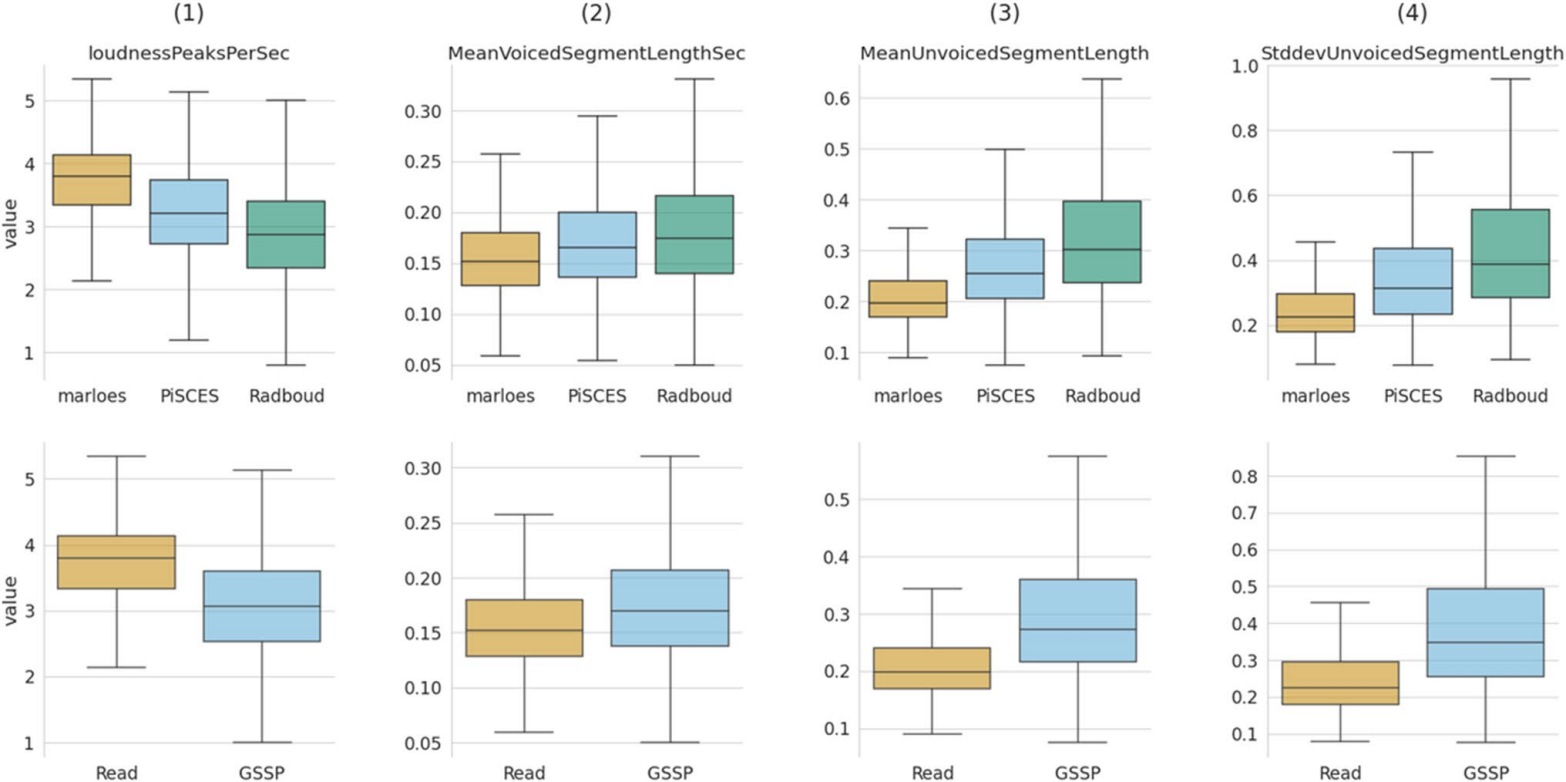
Box plot of temporal features, grouped by collection task (row 1) and speech style (row 2)

**Fig. 7:**
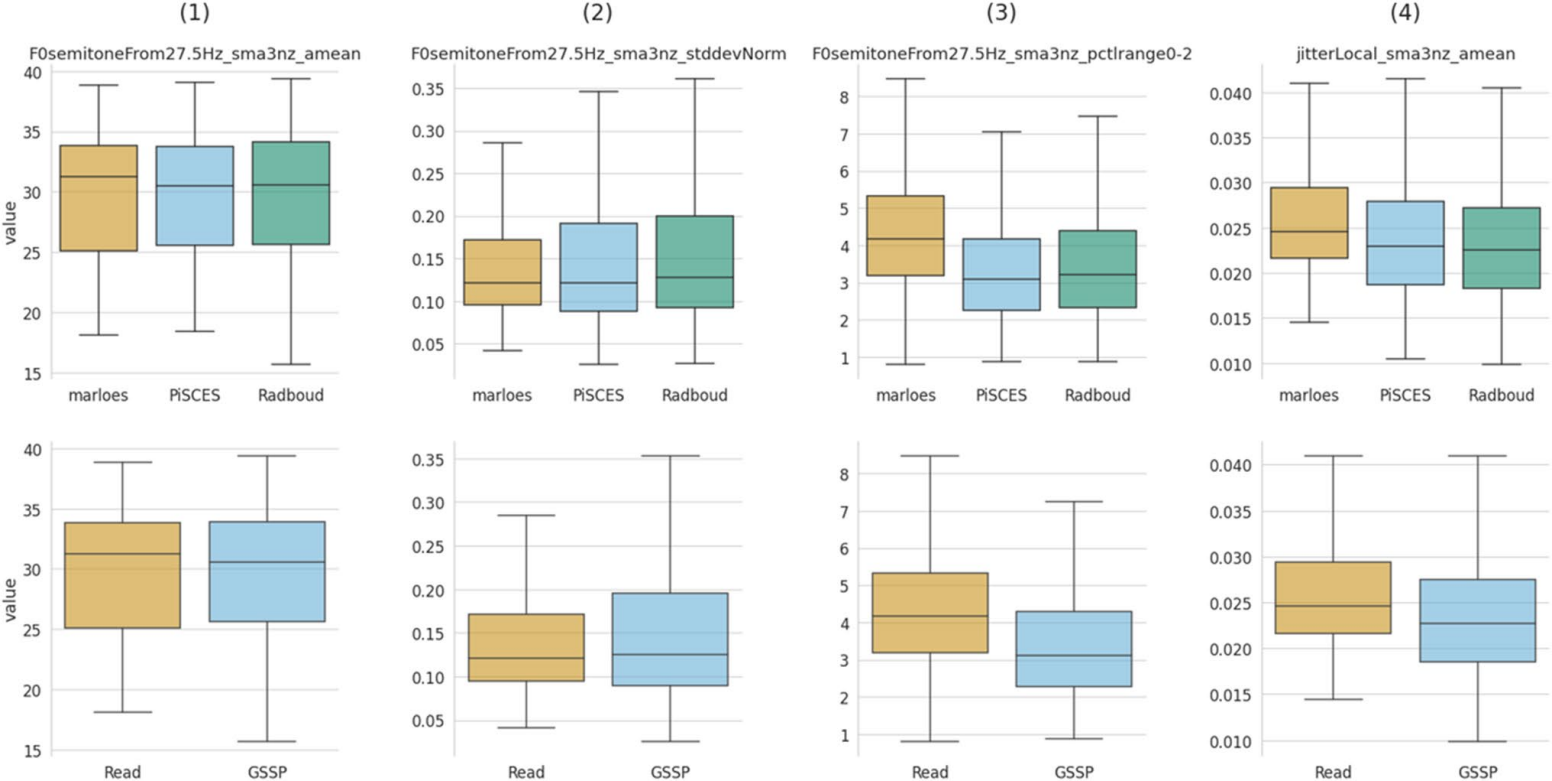
Box plot of frequency-related features, grouped by task (row 1) and speech style (row 2)

**Fig. 8:**
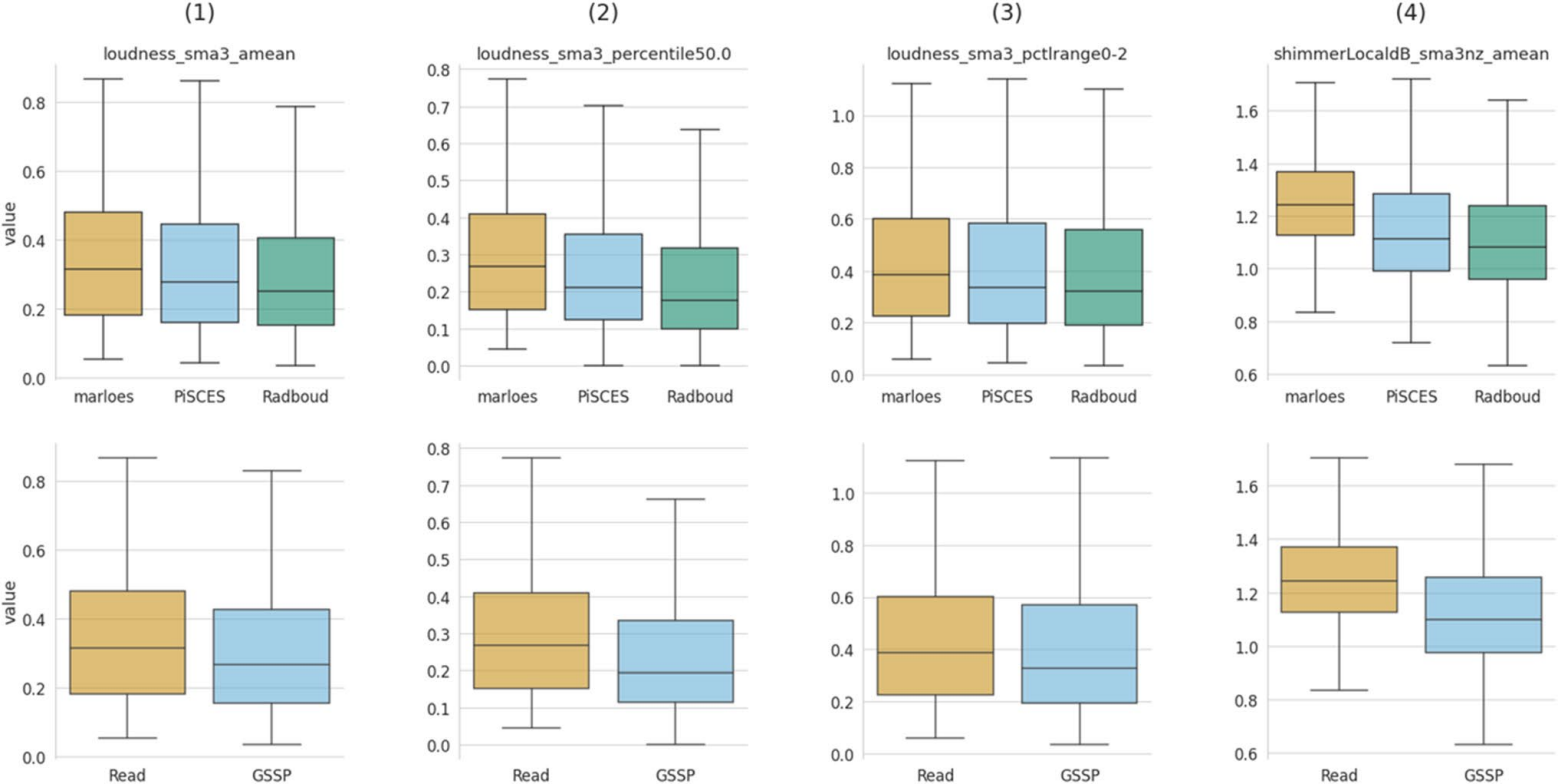
Box plot of amplitude-related features, grouped by task (row 1) and speech style (row 2)

The openSMILE GeMAPSv01b acoustic features were partitioned into three subsets, i.e., a temporal, frequency, and amplitude-related subset. Each subset consists of four distinct features, whose detailed descriptions can be found in Supplemental S4. The visualization of these subsets was conducted using two approaches. The first approach displays the features using a box plot that groups the data on speech collection task [Marloes (M), PiSCES (P), Radboud (R)] and speech style [Read, GSSP (i.e., PiSCES and Radboud)], with each utterance contributing a single data point to the corresponding task (see Figs. 6, 7 and 8). This visualization enables interpretation of the acoustic features in parameter value space. The second approach employs a violin delta-plot, in which utterances of the same participant and speech task are median-aggregated and then subtracted from other speech task aggregations for the same participant, see Figure 14 of Supplemental S6. This results in each participant contributing one data point for each delta. This violin delta plot reveals the distribution shifts and spreads over the various collection tasks. More detailed information regarding the violin delta plot can be found in Supplemental S6.

#### Temporal features

The four temporal features are loudnessPeaksPerSec, MeanVoicedSegmentLengthSec, MeanUnvoicedSegmentLength, and StddevUnvoicedSegmentLength are shown in Fig. 6. Column (1) of Fig. 6 represents the number of loudness peaks, serving as a proxy for syllable rate (Eyben et al., 2016). The coherent distribution shift of the upper and lower subplot of column (1) indicates that the “Marloes” task has a higher articulation rate than both picture description tasks. This observation is consistent with Barik (1977) and Levin et al. (1982), which attributes this lower articulation rate during unscripted speech to the need for planning time when speaking unprepared. Column (2) illustrates the MeanVoicedSegmentLengthSec, which is the distribution of the mean sound duration, indicating slightly shorter voiced segments for the “Marloes” task than for the picture description tasks. This is in line with the notion of voiced segment duration being inversely proportional to the speaking rate [column (1)]. Furthermore, (de Silva et al., 2003) observed a tendency towards longer sound durations for spontaneous speech, which is consistent with our findings. Blaauw (1992) and Laan (1992) found that pauses tend to be more irregular and longer for spontaneous speech, as reflected in the MeanUnvoicedSegmentLength (3) and StddevUnvoicedSegmentLength (4) subplots. Based on these observations, we can conclude that the temporal characteristics of the proposed semi-scripted speech paradigm are trending towards those of unscripted speech.^7^

#### Frequency-related features

Four frequency-related features were utilized, i.e., F0semitoneFrom27.5Hz_sma3nz_amean, F0semitoneFrom27.5Hz_sma3nz_stddevNorm, F0semitoneFrom27.5Hz_sma3nz_pctlrange0-2, and jitterLocal_sma3nz_amean; the mean frequency perturbation. Columns (1) and (2) of Fig. 7 capture the distribution of the fundamental frequency (F0), i.e., its mean and standard deviation respectively. In accordance with de Silva et al. (2003), no clear differences are observed between these acoustic parameters and speech styles. Column (3) visualizes the F0semitoneFrom27.5Hz_sma3nz_pctlrange0-2, which covers the F0-range (i.e., 20th to 80th percentile) and has been reported to be larger in read speech (Batliner et al., 1995), consistent with our findings. Kraayeveld (1997) and Laan (1997) observed more jitter in spontaneous speech, but our findings indicate a significant decrease in jitter (4) for semi-spontaneous speech.

#### Amplitude-related features

Also here, four features have been utilized, i.e., (1) loudness_sma3_amean; the average loudness, (2) loudness_sma_3_percentile50.0; the median loudness, (3) loudness_sma3_pctlrange0-2; the 20th-to-80th percentile loudness range, and (4) shimmerLocaldB_sma3nZ_amean; the mean amplitude perturbation. To date, few results are available regarding loudness parameters and speech style. (Laan, 1992, 1997, p. 1) even applied amplitude normalization to eliminate loudness differences in their experiments. Columns (1) and (2) of Fig. 8 show a slight increase in loudness for the reading task. The loudness range, represented by column (3), is slightly larger for the read-aloud task. We observe a decrease in shimmer (4) for the picture description task, contradicting the findings of (Kraayeveld, 1997; Laan, 1997).

#### Jitter and shimmer inconsistencies

The preceding sections, along with the effect size charts of Supplemental S9, indicated a significant decrease in both jitter and shimmer for the unscripted GSSP task compared to the scripted read-aloud speech. This is in contrast to prior literature that reports the opposite effect, where unscripted speech produces higher jitter and shimmer values than scripted speech. Therefore, we have included this additional section to explore the potential reasons for this inconsistency. Three potential causes for this potential discrepancy are presented below. The first plausible explanation for the acoustic differences could be (1) the nuances in speech styles. The current experiment involved participants being alone in a room and talking to a computer (recording device), while the previous work that produced contrasting results utilized interview based spontaneous speech (Kraayeveld, 1997; Laan, 1997). Therefore, a promising research direction is to investigate the acoustic distinctions between these nuanced speech styles (e.g. monologue vs. conversation, the effect of a study taker on monologue unscripted speech, the effect of the presence of an interviewer in the room). A second potential explanation could be that (2) the openSMILE toolkit may not be capable to accurately extract jitter and shimmer parameters in settings with higher levels of environmental noise. Specifically, sound produced by environmental elements emanating periodic noises such as a (computer) fan could be picked up at the voiced boundaries, i.e., the regions where voicing ends and the environmental elements become more prominent. openSMILE could then start to attribute voiced features on these environmental elements. As detailed in Supplemental S5, abnormally high F0 values were encountered near those voiced boundaries, which largely disappeared when resampling the raw data and adding a small amount of dithering (noise). This supplemental also presents the elevated values observed for the shimmer parameter. Given that read-speech contains a greater proportion of voiced segments, as indicated by the higher syllable rate in Fig. 6(1), there is an increased frequency of voiced boundaries per time unit. This increase in voiced boundaries potentially contributes to the increase in (abnormally high) augmentation in shimmer and jitter values. A third explanation could be that (3) there is indeed a decreasing trend in shimmer and jitter values when analyzing less scripted speech. As outlined in Supplemental S8, a visualization of the weight coefficients of a logistic regression model revealed that a substantial negative coefficient was identified for the shimmer parameter when the model was fitted on either the web app or CGN dataset. Overall, we can conclude that the trend for the majority of acoustic parameters are in accordance with the findings from literature.

#### Acoustic-prosodic validation across image stimuli

**Fig. 9:**
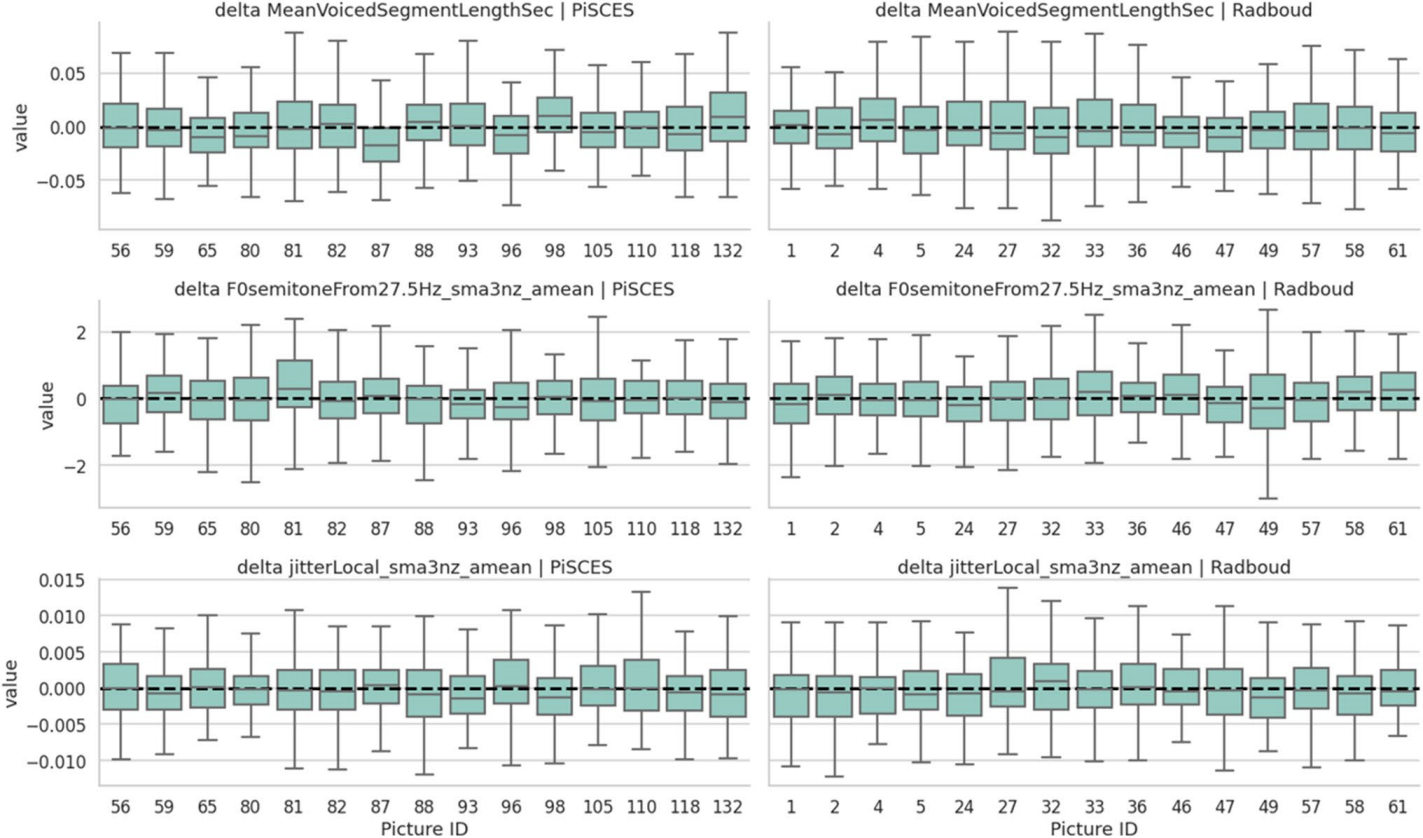
Picture delta box plot of a subset of openSmile features for both the PiSCES (column 1) and Radboud (column 2) image sets. The deltas are calculated by subtracting each value from the participant’s mean for the same DB set

In order to assess the presence of acoustic-prosodic differences across the image stimuli, a delta plot, as outlined in Fig. 9, was created. This figure illustrates the distribution of acoustic features for the utilized images from both the PiSCES and Radboud databases. Notably, aside from picture 87 of PiSCES (pertaining to MeanVoicedSegmentLength), no large deviations are observed compared to other images. Figure 20 of Supplemental S10 portrays a non-participant-normalized version of Fig. 9.

## ECAPA-TDNN projections

**Fig. 10:**
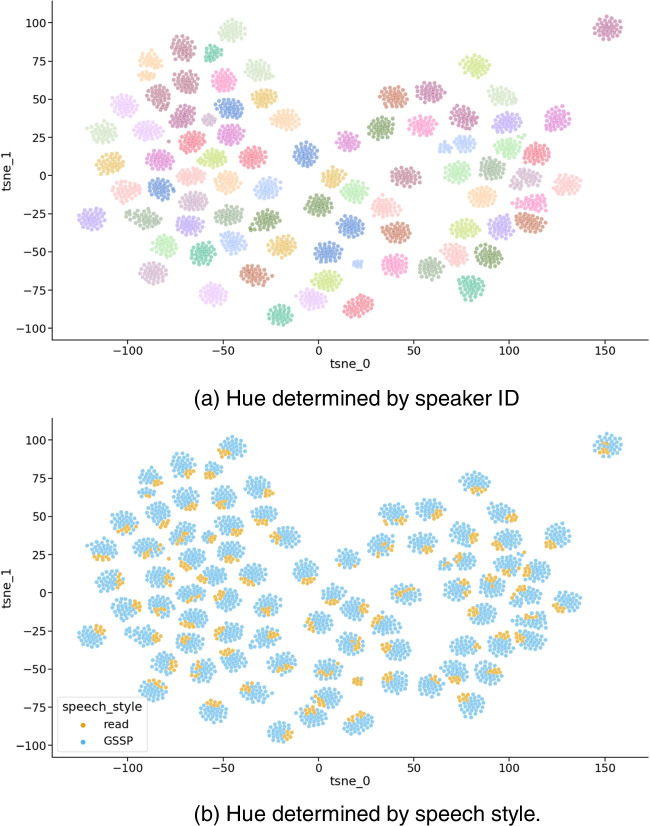
Two-dimensional t-SNE projection of ECAPA-TDNN utterance embeddings. (**a**) Hue determined by speaker ID. (**b**) Hue determined by speech style. *Note.* Each marker represents one speech utterance and, as illustrated by (**a**), each cluster of markers represents utterances by one speaker. When visualizing the colors of each dot based on its speech (trial) style (**b**), we see that generally the individual speech styles cluster together within each speaker's utterances. This hints towards a separability of speech styles based on speaker identification techniques using acoustic properties

In addition to examining the relationship between acoustic-prosodic features in speech styles and positioning this within the literature, we also wanted to investigate speech styles using more data-oriented techniques. To this end, the ECAPA-TDNN architecture (Desplanques et al., 2020) was used to extract fixed-duration embeddings from the utterances. These embeddings were projected into a lower-dimensional space using t-distributed stochastic neighbor embedding (t-SNE, Van der Maaten & Hinton, 2008), the results of which are depicted in Fig. 10. The upper visualization (a) serves as a validation-check, as this demonstrates the primary objective of the ECAPA-TDNN architecture, which is speaker identification. Each cluster consists of a single hue-color, indicating that all cluster points originate from the same user, demonstrating the successful separation of speakers. The lower visualization (b) employs the same projection parameters as (a), but uses speech style as the hue. We observe that in the majority of individual speaker clusters, the “read” speech style utterances are grouped together. This is noteworthy as the primary goal of ECAPA-TDNN is speaker identification, which implies that it has little advantage in utilizing the silent parts of the utterances and primarily focuses on acoustic properties. This observation leads to the hypothesis that the speech style information resides within the captured acoustic properties of the ECAPA-TDNN architecture.

To further validate this claim, a logistic regression model with speech style separability as the objective was fitted on the embeddings. Supplemental S7, Figure 15 illustrates the normality of the embedding features. As such, no further embedding transformations were needed and the features were standardized by removing the mean and scaling to unit variance. The model achieved a balanced accuracy score of 84% ± 1.5% when using fivefold cross-validation with the speaker ID as a grouping variable. Model details can be found in the associated notebook^8^.

## CGN validation

Speech style separability was also assessed using the GeMAPSv01b features. Figure 16 of Supplemental S7 illustrates the distribution of the openSMILE features, which demonstrates a non-normal distribution for most features. As a result, a power transformation was applied as a preprocessing step to ensure more Gaussian-like distributions (Yeo & Johnson, 2000). The GeMAPS model achieved a balanced accuracy score of 83% ± 2.5%, which is comparable to the results obtained from the ECAPA-TDNN model in the above section. A fivefold cross-validation with the speaker ID as the grouping variable was used as the validation setup.

Finally, to ensure maximum generalizability towards the CGN dataset, an educated subset of 24 GeMAPSv01b features was crafted based on their known contribution for speech style representativity. The model achieved a cross-fold score of 81% ± 2%, using the within web app dataset validation setup as described in the previous paragraph. Subsequently, this model was fitted on the whole web application dataset and validated on the external CGN dataset. This resulted in a balanced accuracy score of 70%, as outlined by Table 2. Due to the distribution shift between the training and validation sets (e.g., other recording settings, different demographic groups), a decrease in accuracy compared to the within-web-app cross-fold accuracy was expected. The obtained performance indicates that the GeMAPSv01b web app data speech style decision boundary also holds predictive power when validated on the “B” and “O” components of the CGN dataset, thus indicating an acoustic correspondence between the picture description GSSP speech (web app) and the interviewee speech (CGN). Additional information regarding the model and feature subset selection can be found in the associated notebook^9^.

**Table 2:** CGN validation classification report

	Precision	Recall	F1-score	Support
Read	0.64	0.87	0.74	1643
Unscripted	0.81	0.54	0.65	1714
**accuracy**			0.70	3357
macro_avg	0.73	0.70	0.69	3357
weighted_avg	0.73	0.70	0.69	3357

## Discussion

This paper presents the Ghent Semi-spontaneous Speech Paradigm (GSSP), a picture description task designed to capture speech data for affective-behavioral research in both experimental and real-world settings. The GSSP was developed based on the requirements identified in the field and literature, which were translated to a list of criteria to which the paradigm should adhere to. Specifically, the GSSP was designed to (1) allow for flexible speech recording duration, facilitating convenient incorporation into existing paradigms, (2) present a simple and congruent task, ensuring that the obtained speech is not affected by the load of the speech elicitation method itself, (3) be controllable to limit the inclusion of unwanted latent factors, (4) favor unscripted speech for its prosodic richness and generalizability to everyday speech, and (5) require minimal human effort during data collection to enable use in remote and real-world settings. The GSSP utilizes image stimuli that are emotionally consistent within their respective image set. This enables stimuli randomization in longitudinal designs, which also mitigates learning effects due to familiarity with the stimuli (as occurs with fixed repeated stimuli). Moreover, both image sets are emotionally neutral, limiting confounding effects when implementing the GSSP in known experimental design. Lastly, we specifically designed one image set (PiSCES) to contain stimuli portraying social settings to supply researchers with emotionally neutral, yet congruent stimuli to be used in experimental designs using psychosocial stressors (commonly used, reliable and potent stressors), further limiting confounding effects on stress reactions.

The validation of the GSSP was conducted using a web application that collected speech data from participants. In particular, the participants were instructed to repeatedly perform two tasks; a read-aloud text task and the GSSP. A duration analysis indicated that participants were able to describe images with sufficient duration, therefore adhering to the first criterion.

To provide a correct analysis of the study data, it is important to ensure that only valid speech samples are utilized. Therefore, an essential contribution of this study is the open-source pipeline utilized to process and evaluate speech data, which has been instrumental in ensuring data quality and determining selection criteria. This methodology is not specific to this research and can be applied in other speech data studies, particularly due to its open-source nature.

To analyze the collected data with regard to speech styles, three analyses were performed. The first analysis was concerned with relating acoustic features and existing literature on scripted vs. unscripted speech styles. Acoustic speech features, extracted using the openSMILE GeMAPSv01b functional configuration, exhibited a trend that is consistent with literature on the targeted speech styles, i.e., scripted read-aloud speech and unscripted spontaneous speech, therefore adhering to the fourth requirement. Nonetheless, the observed trend was not consistent across all the analyzed features. Specifically, the jitter (our fourth frequency-related feature) and the shimmer values (our fourth amplitude-related feature) did not align with existing literature in this field. Jitter and shimmer both were lower for the unscripted GSSP task compared to the scripted read-aloud speech, which contradicts literature that reports the opposite effect. This discrepancy can potentially be attributed to (a combination of) three reasons, which are thoroughly discussed in openSMILE acoustics section.

The second analysis is concerned with data driven techniques. Specifically, the ECAPA-TDNN t-SNE projection, presented in Fig. 10, demonstrated that speaker clusters are further sub-grouped according to speech style. A speech style separability experiment on the web app data, utilizing the GeMAPSv01b features, yielded a balanced accuracy of 83%, which is in agreement with the findings of Levin et al. (1982), who reported that listeners were able to distinguish between spontaneous and read-aloud speech with an accuracy of 84%, primarily based on temporal characteristics and false starts.

The third analysis assessed the generalization of the web app speech style separability by performing an out-of-dataset validation on the CGN dataset, using scripted read-aloud speech (comp. O) and spontaneous interviewee speech (comp. B). This validation resulted in a lower, but still satisfactory, balanced accuracy score of 70%. These results indicate that there is a clear separation between speech from the read-aloud and GSSP task, and that the acoustic properties of the GSSP task are in accordance with those of spontaneous speech from well-regarded databases. Future research should explore how the GSSP compares to speech styles other than read-aloud speech. While we used the web application dataset to examine the GSSP's acoustic properties via repeated measures within a session, the primary goal of the paradigm is continuous monitoring. Therefore, subsequent studies should evaluate the GSSP across different sessions. Moreover, while our study effectively contrasts read versus semi-spontaneous speech, it does not isolate the impact of repetition present in Marloes but absent in GSSP, a distinction that requires further investigation.

In our analyses, we applied a 15-second duration criterion to facilitate comparisons between the GSSP and Marloes samples. Moreover, we focused our analyses on the latter part of the utterances to minimize the influence of similar starting sessions caused by the repetitive nature of our GSSP collection procedure. It is important to note that this choice of duration and the emphasis on end-of-utterance data are not rigid requirements, but rather decisions informed by the specific design of our study. Therefore, it is advisable for future studies utilizing the GSSP to adjust their duration criteria and analysis window positioning to suit their specific objectives and study design.

The significant variation in (quality of) utilized recording devices, introduced some degree of compromise to the validity of the analysis. Future studies that employ this paradigm are advised to implement stricter guidelines to limit the inclusion of unwanted variables (third criterion). Despite this limitation, the web application demonstrated the ability to deploy the GSSP at scale (fifth criterion) by needing no human interference during collection. Furthermore, the unscripted nature (fourth criterion) of this paradigm presents an opportunity to explore semantic-content aspects, as previous research has established the potential of these modalities as markers for various disorders (de Boer et al., 2020; Mueller et al., 2018).

In conclusion, the GSSP demonstrates qualities of intuitiveness, scalability, accessibility, and brevity (i.e., 30–60 seconds), making it a suitable addition to well-established experimental studies for collecting unscripted speech during key moments, such as before and after exposure to stressors or emotional loads. This approach does not compromise other essential outcome variables and can be seamlessly integrated into remote-sensing applications, facilitating research on longitudinal mental well-being using speech and mood correlates (Kappen et al., 2023). We hypothesize that findings obtained from utilizing the GSSP will be easier translatable to real-world settings, such as speech collected in team or board meetings, presentations, or any other social setting. This research aligns with the conclusion from Xu (2010), which states that employed speech elicitation techniques need constant updates to gain increasingly better insights into the full complexity of speech. We are convinced that our presented GSSP, supported by the documented code, data^10^, and analysis results, enable behavioral researchers to incorporate an unscripted picture description task in their research studies. Future work should focus on further assessing the nuances in speech styles and investigating environmental effects on (this) paradigm(s), such as the presence of a study taker.

### Supplementary information

ESM 1 (PDF 6345 kb)

## Data Availability

- All data and code are publicly available at https://www.kaggle.com/datasets/jonvdrdo/gssp-web-app-data - The code is available as well on GitHub at: - https://github.com/predict-idlab/gssp_analysis - https://github.com/predict-idlab/gssp_web_app

## Footnotes

1 https://github.com/predict-idlab/gssp_analysis, https://github.com/predict-idlab/gssp_web_app

2 https://www.kaggle.com/datasets/jonvdrdo/gssp-web-app-data

3 https://github.com/predict-idlab/gssp_web_app

4 https://github.com/predict-idlab/gssp_analysis

5 We conducted an acoustic analysis on the duration of the entire utterance, and found that the results were consistent with those obtained from the last 15 seconds of voiced data for both the ECAPA-TDNN projections and openSMILE features.

6 https://github.com/predict-idlab/gssp_analysis/scripts/1.2_FactorAnalysis.pdf

7 We also observe that the speech rate is lower and the pauses are longer for the Radboud task compared to PiSCES, which might be caused by the homogeneity of the Radboud images, making it substantially harder to describe novel things.

8 gssp_analysis/notebooks/0.6_ECPA_TDNN_npy.ipynb

9 gssp_analysis/notebooks/1.3_OpenSMILE_ML.ipynb

10 The provided web app dataset can also be used to analyze acoustic effects of repetitive reading, as participants read the same (phonetically balanced) text nine times.
