# Supplementary material for: Ecologically valid speech collection in behavioral research: The Ghent Semi-spontaneous Speech Paradigm (GSSP)
Source: Behav Res Methods. 2023 Dec 13;56(6):5693–708. doi: 10.3758/s13428-023-02300-4 (PMC11335842; doi:10.3758/s13428-023-02300-4)
Supplement: Supplementary file 1 — (PDF 6345 kb) [file 13428_2023_2300_MOESM1_ESM.pdf]

Supplementary Materials

For

Ecologically Valid Speech Collection in Behavioral Research: The Ghent Semi-spontaneous Speech Paradigm (GSSP)

Jonas Van Der Donckt, Mitchel Kappen, Vic Degraeve, Kris Demuynck,
Marie-Anne Vanderhasselt, Sofie Van Hoecke

Contents:

S1. Web Application Details
- S1.1. Welcome Page
- S1.2. Introduction Page
- S1.3. Instruction Page
- S1.4. Rest Block
- S1.5. “Marloes” Text
- S1.6. GSSP Web App Image Subsets

S2. Speech Data Parsing
S3. Number of Removed Recordings per Participant
S4. OpenSMILE Feature Subset
S5. OpenSMILE Sampling Rate Inconsistency
S6. OpenSMILE Delta Visualizations
S7. ECAPA-TDNN & GeMAPS Distribution Plots
S8. Logistic Regression Weight Coefficients
S9. Effect Size Shimmer & Jitter
- S9.1. Shimmer
- S9.2. Jitter

S10 Image stimuli Analysis
S11. Session Number Analysis

S1. Web Application Details

S1.1. Welcome Page

Figure 1

Welcome page Screenshot.

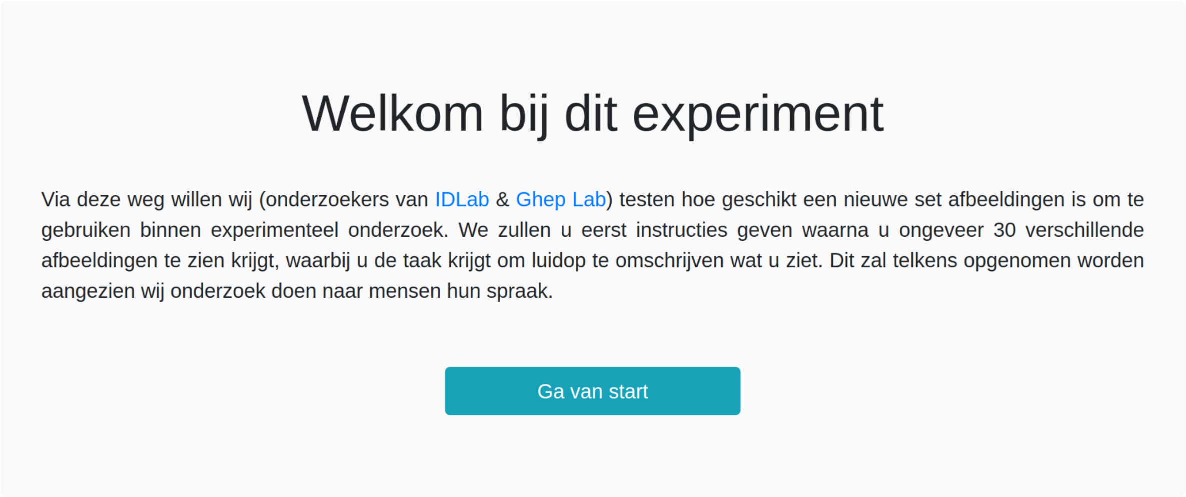

S1.2. Introduction Page

Original instructions:

- Deze taak is enkel uit te voeren op een laptop/computer.
- Gelieve uw oortelefoons/koptelefoon of iets dergelijks te gebruiken met microfoon; dit zorgt voor hoge kwaliteit opnames.
- Indien u zeker bent dat de microfoon van uw computer van voldoende hoge kwaliteit is, mag u deze gebruiken.
- Zorg dat u plaatsneemt in een rustige omgeving waar u 30 minuten omringt wordt door zo min mogelijk afleiding en geluid.
- Zorg dat u een glas water bij uw laptop/computer hebt staan.
- Tijdens de taak zitten een aantal korte drinkpauzes zodat u geen droge keel krijgt door het veelvuldig praten – ook dit zorgt voor hoge kwaliteit opnames.

Translated instructions:

- This task is only to be performed on a desktop.

- We strongly suggest using a headphone, only use your desktops' microphone when you are sure that the recording quality of the device is high.
- Make sure that you are in a quiet and distraction free environment for at least 30 minutes.
- Make sure that you have a glass of water next to your desktop
- During the task, several drinking pauses will occur. This ensures that you will not suffer from a dry throat while speaking.

Figure 2

Introduction page screenshot.

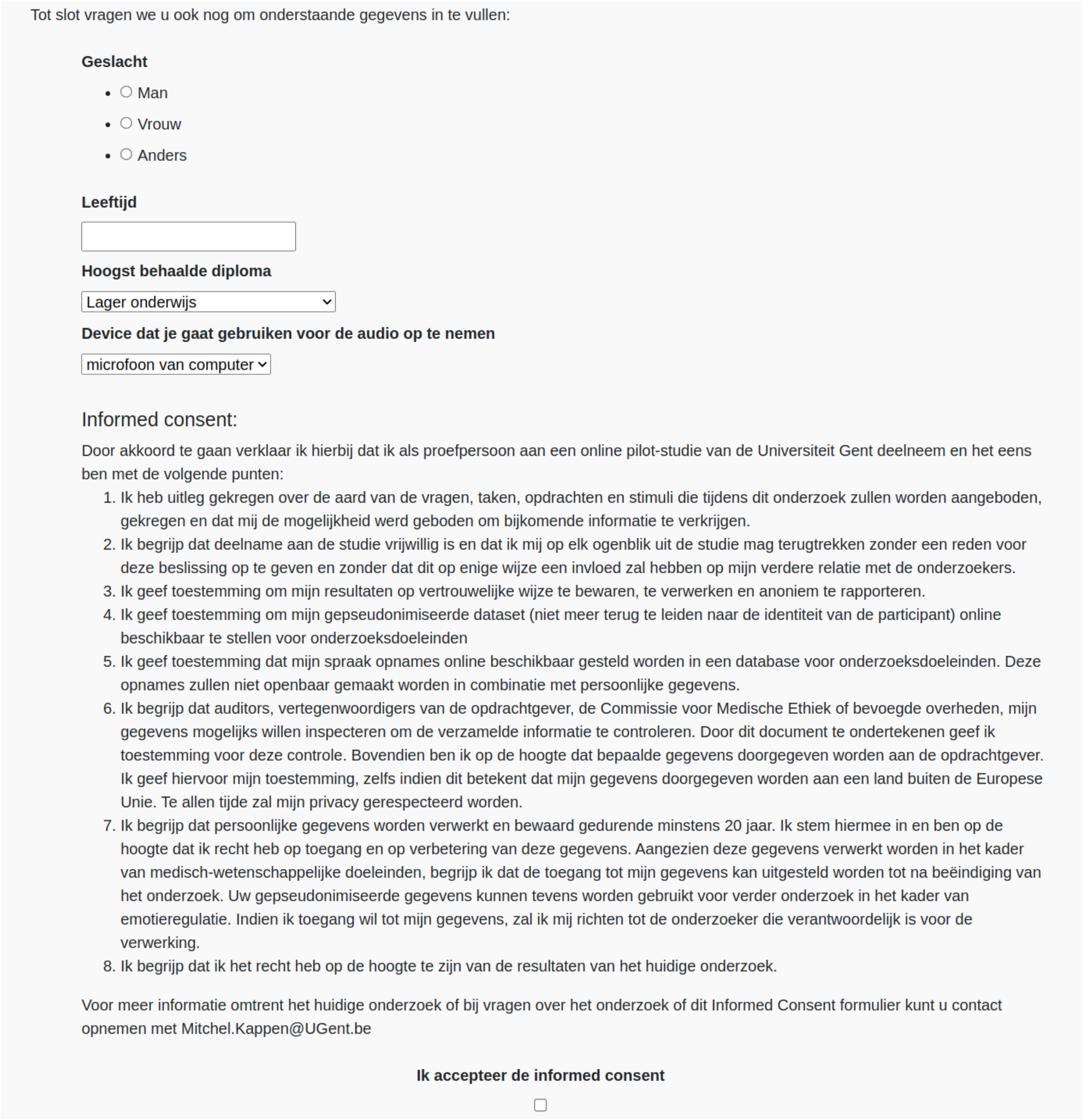

Tot slot vragen we u ook nog om onderstaande gegevens in te vullen:

Geslacht

- ○ Man
- ○ Vrouw
- ○ Anders

Leeftijd

Hoogst behaalde diploma

Lager onderwijs

Device dat je gaat gebruiken voor de audio op te nemen

microfoon van computer

Informed consent:

Door akkoord te gaan verklaar ik hierbij dat ik als proefpersoon aan een online pilot-studie van de Universiteit Gent deelneem en het eens ben met de volgende punten:

1. Ik heb uitleg gekregen over de aard van de vragen, taken, opdrachten en stimuli die tijdens dit onderzoek zullen worden aangeboden, gekregen en dat mij de mogelijkheid werd geboden om bijkomende informatie te verkrijgen.
2. Ik begrijp dat deelname aan de studie vrijwillig is en dat ik mij op elk ogenblik uit de studie mag terugtrekken zonder een reden voor deze beslissing op te geven en zonder dat dit op enige wijze een invloed zal hebben op mijn verdere relatie met de onderzoekers.
3. Ik geef toestemming om mijn resultaten op vertrouwelijke wijze te bewaren, te verwerken en anoniem te rapporteren.
4. Ik geef toestemming om mijn gepseudonimiseerde dataset (niet meer terug te leiden naar de identiteit van de participant) online beschikbaar te stellen voor onderzoeksdoeleinden
5. Ik geef toestemming dat mijn spraak opnames online beschikbaar gesteld worden in een database voor onderzoeksdoeleinden. Deze opnames zullen niet openbaar gemaakt worden in combinatie met persoonlijke gegevens.
6. Ik begrijp dat auditors, vertegenwoordigers van de opdrachtgever, de Commissie voor Medische Ethiek of bevoegde overheden, mijn gegevens mogelijks willen inspecteren om de verzamelde informatie te controleren. Door dit document te ondertekenen geef ik toestemming voor deze controle. Bovendien ben ik op de hoogte dat bepaalde gegevens doorgegeven worden aan de opdrachtgever. Ik geef hiervoor mijn toestemming, zelfs indien dit betekent dat mijn gegevens doorgegeven worden aan een land buiten de Europese Unie. Te allen tijde zal mijn privacy gerespecteerd worden.
7. Ik begrijp dat persoonlijke gegevens worden verwerkt en bewaard gedurende minstens 20 jaar. Ik stem hiermee in en ben op de hoogte dat ik recht heb op toegang en op verbetering van deze gegevens. Aangezien deze gegevens verwerkt worden in het kader van medisch-wetenschappelijke doeleinden, begrijp ik dat de toegang tot mijn gegevens kan uitgesteld worden tot na beëindiging van het onderzoek. Uw gepseudonimiseerde gegevens kunnen tevens worden gebruikt voor verder onderzoek in het kader van emotieregulatie. Indien ik toegang wil tot mijn gegevens, zal ik mij richten tot de onderzoeker die verantwoordelijk is voor de verwerking.
8. Ik begrijp dat ik het recht heb op de hoogte te zijn van de resultaten van het huidige onderzoek.

Voor meer informatie omtrent het huidige onderzoek of bij vragen over het onderzoek of dit Informed Consent formulier kunt u contact opnemen met Mitchel.Kappen@UGent.be

Ik accepteer de informed consent

S1.3. Instruction Page

Figure 3-5

Instruction page screenshots.

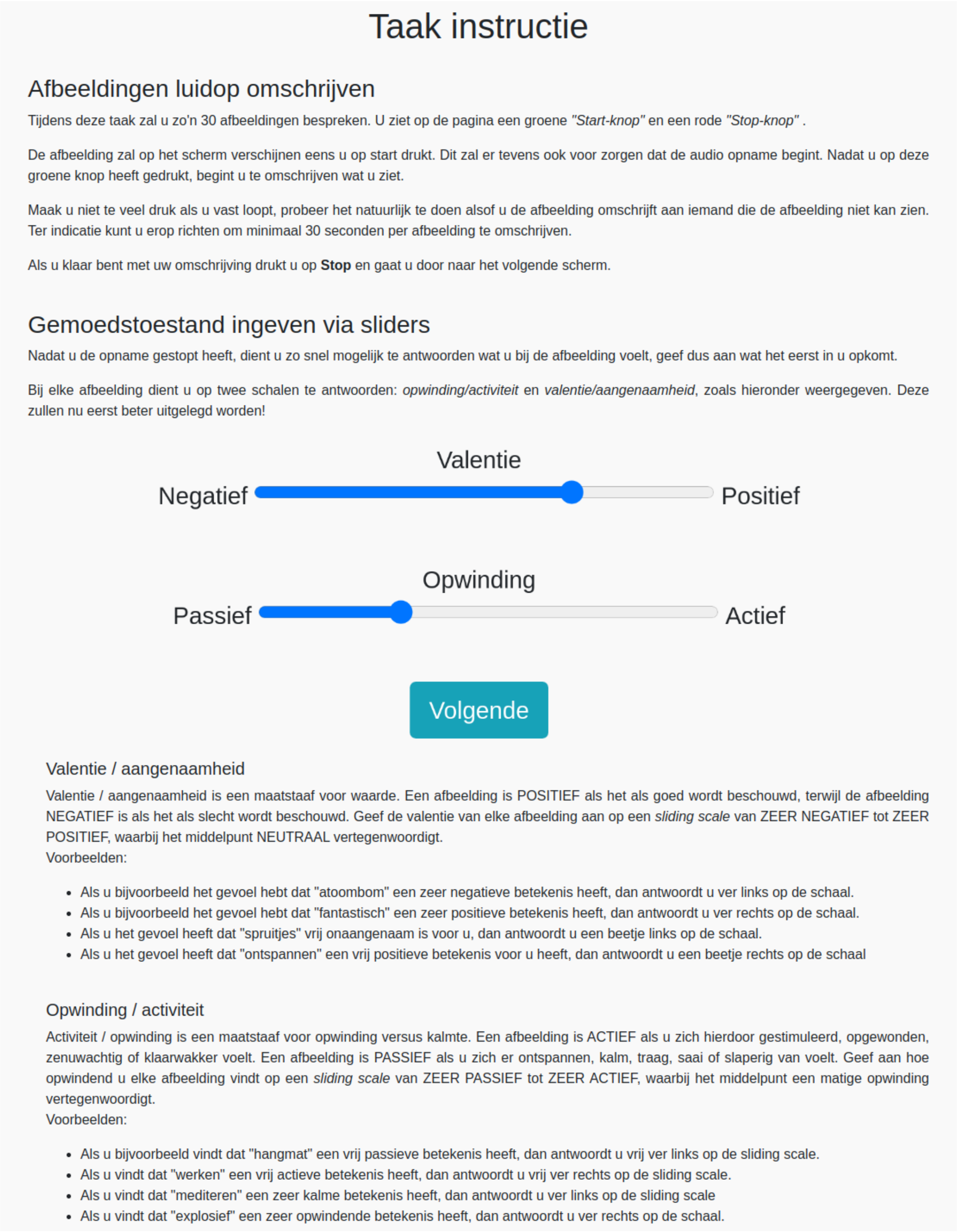

Taak instructie

Afbeeldingen luidop omschrijven

Tijdens deze taak zal u zo'n 30 afbeeldingen bespreken. U ziet op de pagina een groene *"Start-knop"* en een rode *"Stop-knop"* .

De afbeelding zal op het scherm verschijnen eens u op start drukt. Dit zal er tevens ook voor zorgen dat de audio opname begint. Nadat u op deze groene knop heeft gedrukt, begint u te omschrijven wat u ziet.

Maak u niet te veel druk als u vast loopt, probeer het natuurlijk te doen alsof u de afbeelding omschrijft aan iemand die de afbeelding niet kan zien. Ter indicatie kunt u erop richten om minimaal 30 seconden per afbeelding te omschrijven.

Als u klaar bent met uw omschrijving drukt u op **Stop** en gaat u door naar het volgende scherm.

Gemoedstoestand ingeven via sliders

Nadat u de opname gestopt heeft, dient u zo snel mogelijk te antwoorden wat u bij de afbeelding voelt, geef dus aan wat het eerst in u opkomt.

Bij elke afbeelding dient u op twee schalen te antwoorden: *opwinding/activiteit* en *valentie/aangenaamheid*, zoals hieronder weergegeven. Deze zullen nu eerst beter uitgelegd worden!

Valentie / aangenaamheid

Valentie / aangenaamheid is een maatstaaf voor waarde. Een afbeelding is POSITIEF als het als goed wordt beschouwd, terwijl de afbeelding NEGATIEF is als het als slecht wordt beschouwd. Geef de valentie van elke afbeelding aan op een *sliding scale* van ZEER NEGATIEF tot ZEER POSITIEF, waarbij het middelpunt NEUTRAAL vertegenwoordigt.
Voorbeelden:

- Als u bijvoorbeeld het gevoel hebt dat "atoombom" een zeer negatieve betekenis heeft, dan antwoordt u ver links op de schaal.
- Als u bijvoorbeeld het gevoel hebt dat "fantastisch" een zeer positieve betekenis heeft, dan antwoordt u ver rechts op de schaal.
- Als u het gevoel heeft dat "spruitjes" vrij onaangenaam is voor u, dan antwoordt u een beetje links op de schaal.
- Als u het gevoel heeft dat "ontspannen" een vrij positieve betekenis voor u heeft, dan antwoordt u een beetje rechts op de schaal

Opwinding / activiteit

Activiteit / opwinding is een maatstaaf voor opwinding versus kalmte. Een afbeelding is ACTIEF als u zich hierdoor gestimuleerd, opgewonden, zenuwachtig of klaarwakker voelt. Een afbeelding is PASSIEF als u zich er ontspannen, kalm, traag, saai of slaperig van voelt. Geef aan hoe opwindend u elke afbeelding vindt op een *sliding scale* van ZEER PASSIEF tot ZEER ACTIEF, waarbij het middelpunt een matige opwinding vertegenwoordigt.
Voorbeelden:

- Als u bijvoorbeeld vindt dat "hangmat" een vrij passieve betekenis heeft, dan antwoordt u vrij ver links op de sliding scale.
- Als u vindt dat "werken" een vrij actieve betekenis heeft, dan antwoordt u vrij ver rechts op de sliding scale.
- Als u vindt dat "mediteren" een zeer kalme betekenis heeft, dan antwoordt u ver links op de sliding scale
- Als u vindt dat "explosief" een zeer opwindende betekenis heeft, dan antwoordt u ver rechts op de schaal.

Er zullen afbeeldingen uit twee verschillende sets worden aangeboden, ofwel gezichten, ofwel tekeningen van bepaalde settings. Hieronder vindt u twee video's (1 voor elk van de twee sets) met hoe een trial eruit ziet: Start -> afbeelding omschrijven -> Stop -> Beoordelen.

In deze video's ziet u een timer eronder om een indicatie te krijgen van hoe lang deze omschrijving is. Tijdens uw taak hebben we graag dat u minimaal zo'n 30 seconden omschrijft maar zult u geen timer zien. Maak u niet te veel druk mocht u vastlopen.
Kleine opmerking: De blauwe "Volgende" knop bij het ingeven van de gemoedstoestand wordt slechts zichtbaar eens het spraaksegment succesvol naar de server doorgestuurd is!

Gezicht

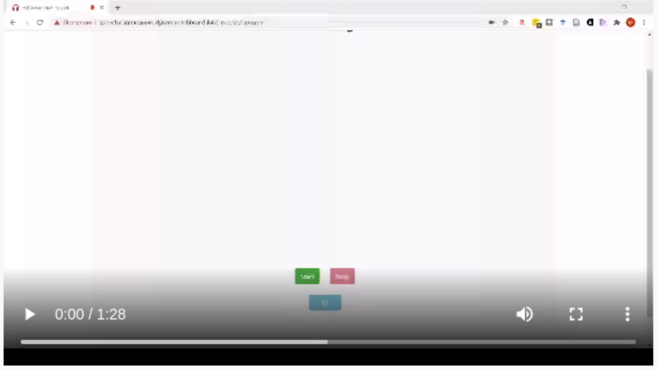

Tekening

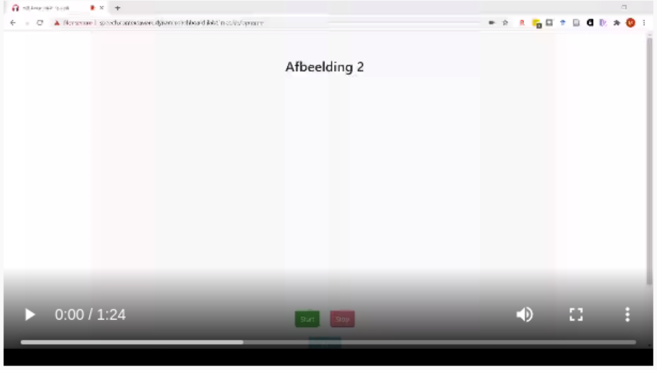

Tekst voorlezen

Tussen de afbeeldingen door krijgt u soms de instructie om een tekstje voor te lezen. Hiervoor wordt hetzelfde principe als bij de afbeeldingen toegepast; *groene start-knop -> tekst verschijnt -> voorlezen -> rode stop-knop*. De vooraf bepaalde tekst die u zal voorlezen, zal steeds de volgende zijn; Graag vragen we u om deze nu al eens hardop voor te lezen:

Papa en Marloes staan op het station.
Ze wachten op de trein.
Eerst hebben ze een kaartje gekocht.
Er stond een hele lange rij, dus dat duurde wel even.
Nu wachten ze tot de trein eraan komt.
Het is al vijf over drie, dus het duurt nog vier minuten.
Er staan nog veel meer mensen te wachten.
Marloes kijkt naar links, in de verte ziet ze de trein al aankomen.

Na het opnemen van deze tekst, zal opnieuw gevraagd worden, om uw gemoedstoestand in te geven via de sliders. Als extra illustratie van dit proces kan u naar onderstaande video kijken.

Tekst voorlezen

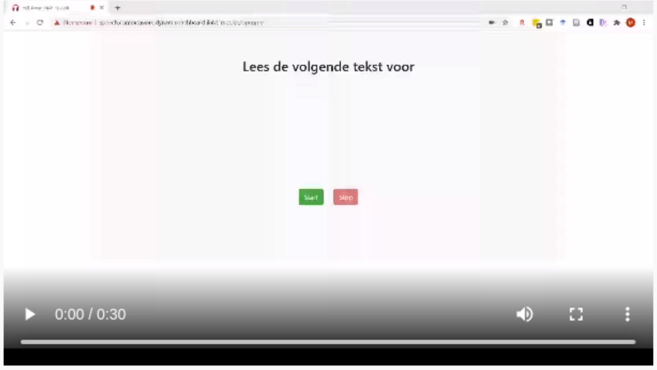

Pauzes

Ook krijgt u soms de instructie om een slok water te nemen. Doe dit zeker, aangezien uw keel anders uitdroogt, wat onprettig is en de opname beïnvloedt.

Tot slot willen we u vragen om de audiokwaliteit van uw microfoon te testen. Hiervoor drukt u op start, spreekt u iets in, waarna u de opname kan beluisteren door op pauze te drukken.

Eens dit werkt, en u op de blauwe knop heeft gedrukt, zal u doorverwezen worden naar een **blanco-pagina** die u voor 5 minuten te zien krijgt, het is de bedoeling voor deze periode om uw ogen te sluiten en op de ademhaling te focussen, zodat u helemaal tot rust komt. Eens deze periode voorbij is zal u een toon horen.

Succes!!

Test uw audiokwaliteit:

Start Stop Play

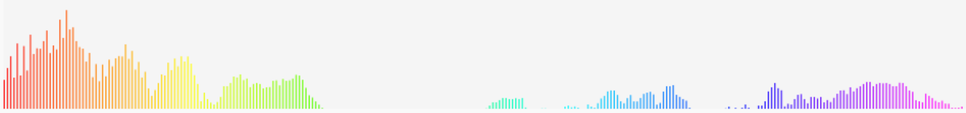

Verder naar rustmoment

S1.4. Rest Block

Figure 6

Rest block screenshot.

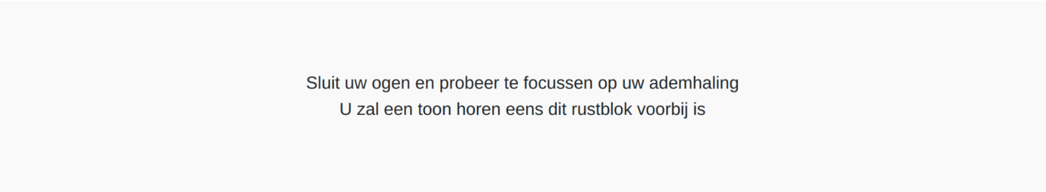

S1.5. “*Marloes”* Text

Papa en Marloes staan op het station.
Ze wachten op de trein.
Eerst hebben ze een kaartje gekocht.
Er stond een hele lange rij, dus dat duurde wel even.
Nu wachten ze tot de trein eraan komt.
Het is al vijf over drie, dus het duurt nog vier minuten.
Er staan nog veel meer mensen te wachten.
Marloes kijkt naar links, in de verte ziet ze de trein al aankomen.

S1.6. GSSP Web App Image Subsets

Figure 7

PiSCES image subset.

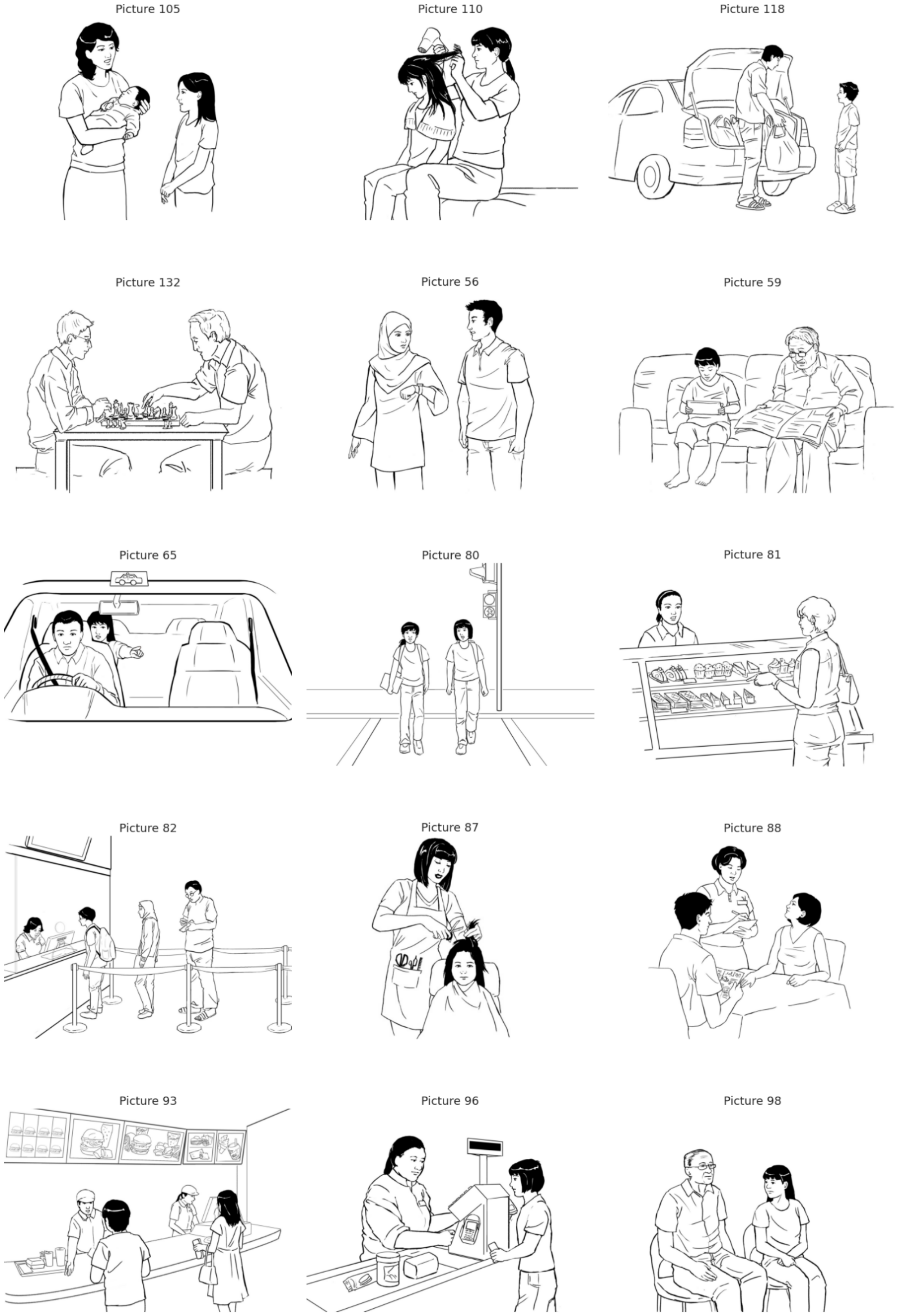

Figure 8

Radboud faces image subset.

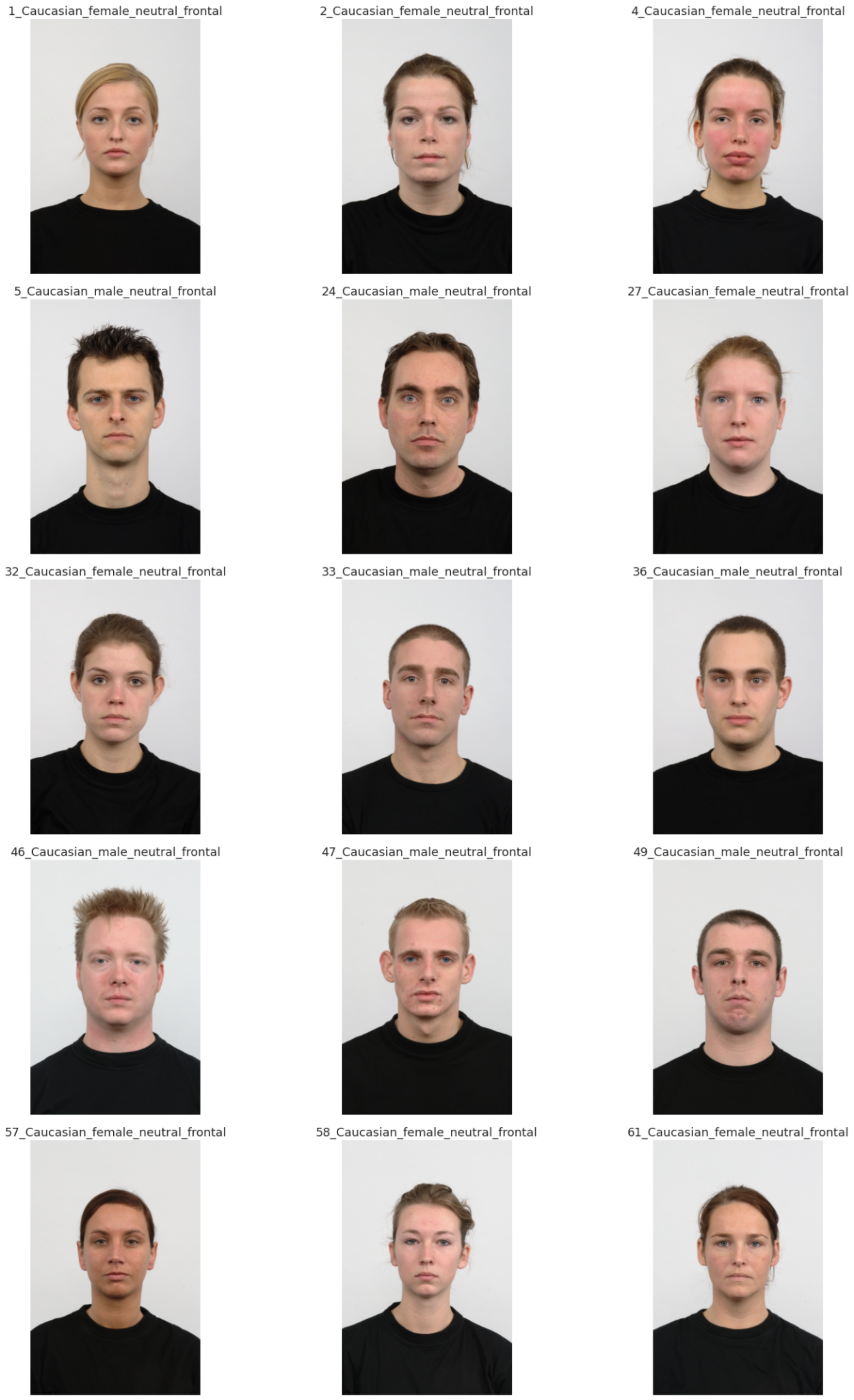

S2. Speech Data Parsing

Figure 9

Visualizations employed during the participant audio analysis step.

```
analyze_audio_quality(
    df_session=df_session,
    ID=np.unique(df_session.ID)[row - 2],
    **numb_dict,
    plot=True,
    audio=True,
    norm_audio=True,
    vad=True,
    show_wav_features=False,
    noise_reduction=False,
    audio_begin_s=5,
    audio_end_s=10,
    smile_lld=smile_lld,
    feat_cols=feat_cols,
)
```

```
age-sex: 30 - female            education: ho          device: pc-mic
#Radboud: 15   -   #Pisces: 15   -   #Marloes: 7   -   SR=48000Hz
```

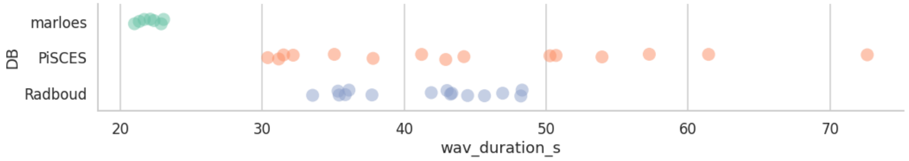


```
============================== PiSCES ==============================
Playing from 5 to 10 seconds  |orig norm| 16khz norm |
```

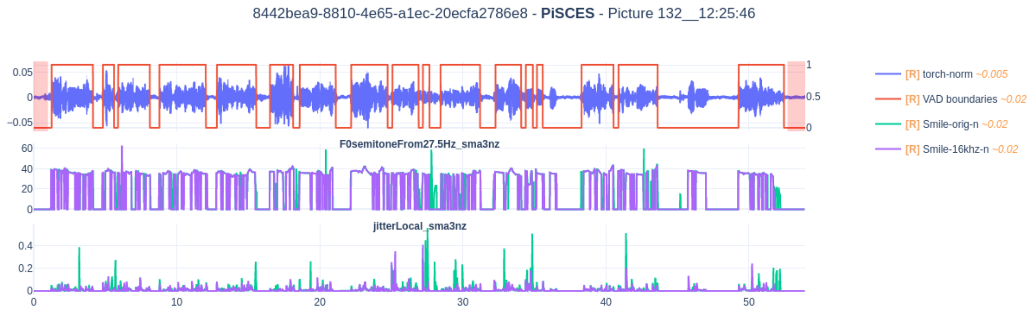


```
Playing from 5 to 10 seconds  |orig norm| 16khz norm |
```

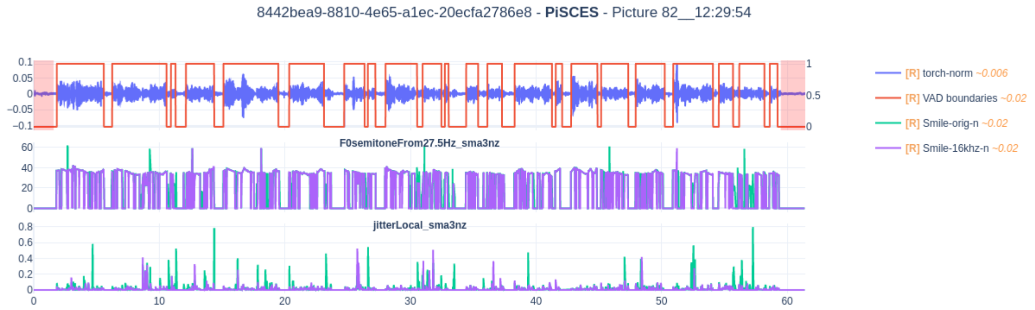

	norm_16Khz__PiSCES_10_23	norm_16Khz__PiSCES_12_28
**Loudness_sma3**	1.000000	1.000000
**alphaRatio_sma3**	0.999000	0.999000

Note. The upper plot highlights the recorded, non-VAD cropped, utterance duration for each database subset, allowing to detect duration outliers. Below this duration plot, two utterances from the PiSCES subset are analyzed. For each utterance, the raw and transformed audio can be listened to. Below the audio players, a time-series visualization highlights the predictions of a voice activity detection (VAD) model and the extracted openSMILE Low Level Descriptors (LLDs). The VAD predictions are used to detect the first and last speech segments, which on its end determine the regions that will be omitted in the parsing block, i.e., the red shaded areas on the upper subplot. The purpose of the two lower subplots is to assess the ability of OpenSMILE to qualitatively extract speech metrics from the excerpts. The chosen metrics, fundamental frequency (F0) and jitter, are useful indicators of the stability of the feature extraction process. Finally, the table at the bottom of the figure shows the correlation of the extracted speech features with respect to the raw (non-resampled) WAV file. The visualization code can be found here[1].

Figure 10

Manual inspection of a participant with a large silent part at the end.

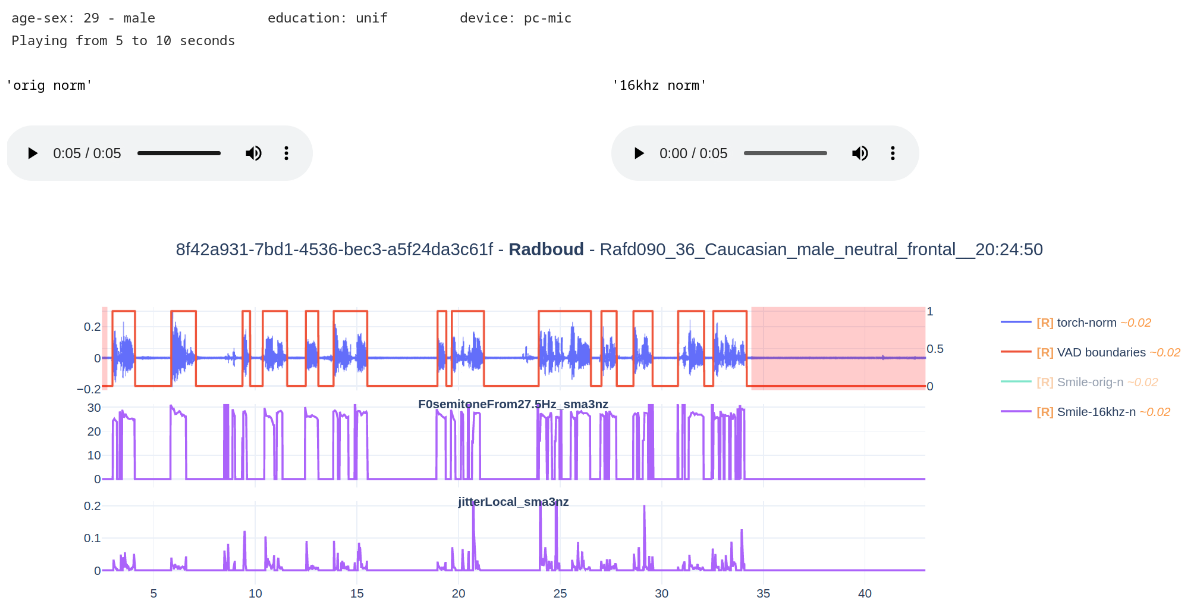

Note. The Voice Activity Detection (VAD) segmentation is able to detect this silence. The red-shaded rectangle indicates that this part will not be included in the parsed segment.

[1] https://github.com/predict-idlab/gssp_analysis/notebooks/0.3_Process_audio_Analyze_quality.ipynb

S3. Number of Removed Recordings per Participant

Figure 11

Illustration of number of removed recordings per participant by not meeting the 15-second voiced duration criteria. The distribution reveals that for 75 of the 82 participants, fewer than 4 recordings are removed. This visualization also indicates that the majority of excluded recordings can be attributed to a small group of participants (i.e., 3-7 participants).

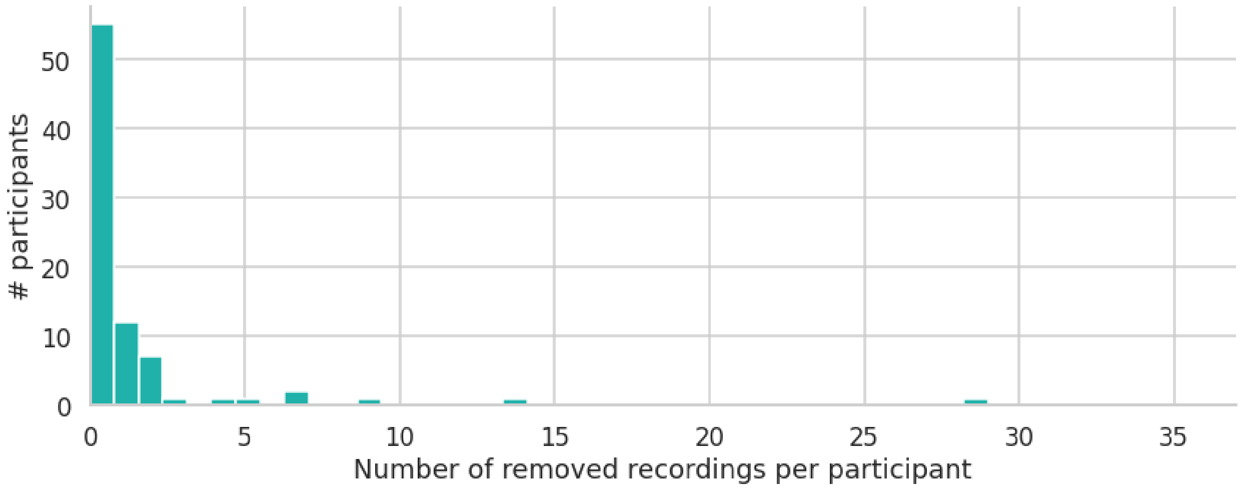

S4. OpenSMILE Feature Subset

Table 1

Description of utilized OpenSMILE GeMAPSv01b Functional features.

GeMAPSv01b name	Description
**Temporal**	
loudnessPeaksPerSec	The mean rate of loudness peaks, i.e., the number of loudness peaks per second.
MeanVoicedSegmentLengthSec	The mean length of continuously voiced regions (F0 > 0).
MeanUnvoicedSegmentLength<br>StddevUnvoicedSegmentLength	The mean length and the standard deviation of unvoiced regions (i.e., F0 = 0; approximating pauses).
**Spectral**	
F0semitoneFrom27.5Hz_sma3nz_amean<br>F0semitoneFrom27.5Hz_sma3nz_stddevNorm<br>F0semitoneFrom27.5Hz_sma3nz_pctlrange0-2	Aggregation of moving windows in which the Logarithmic F0 is computed on a semitone frequency scale, starting at 27.5 Hz (semitone 0). The moving window output is first smoothened by a moving average with a window size of 3 that only includes non-zero values (sma3nz). The aggregations are respectively: mean, standard deviation, and range of 20th to 80th percentile.<br>To convert the semitone frequency ($F0_{st}$) to hertz, the following formula can be applied:<br>$$F0_{Hz} = 27.5Hz * 2^{F0_{st}/12}$$
jitterLocal_sma3nz_amean	Mean aggregation of moving window in which the deviation of individual consecutive F0 period lengths is computed.
**Amplitude**	
loudness_sma3_amean<br>loudness_sma3_percentile50.0<br>loudness_sma3_pctlrange0-2	Aggregation of moving windows of perceived signal intensity from an auditory spectrum. The aggregation are respectively: mean, median, and range of 20th to 80th percentile.
shimmerLocaldB_sma3nz_amean	Mean aggregation of moving windows of the differences of the peak amplitudes of consecutive F0 periods.

Note. We further refer to Appendix 6.1 of (Eyben et al., 2016) for implementation details.

S5. OpenSMILE Sampling Rate Inconsistency

Figure 12

Illustration of inconsistency in the GeMAPSv01b Low-Level-Descriptors (LLDs) values when varying the sample frequency. The selection of F0semitone and jitterLocal as features was based on prior utilization in the current work and their interpretability

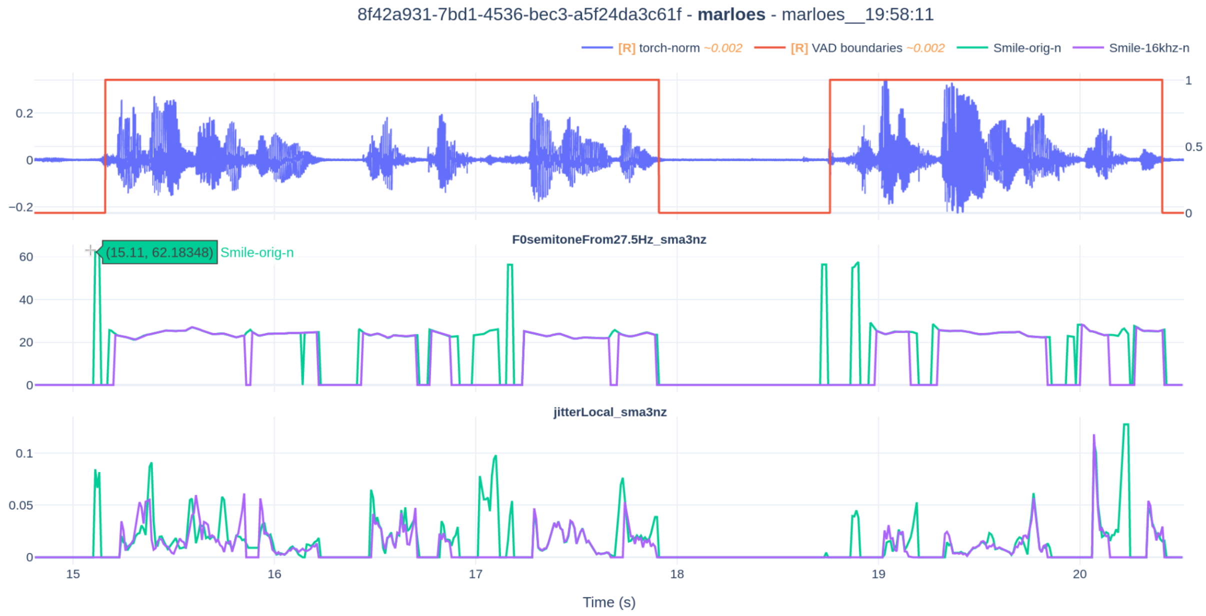

The above figure demonstrates the instability of the GeMAPSv01b Low-Level-Descriptors (LLDs) when the audio sample rate is altered. At approximately second 15 and 17, the *F0semitone* and *jitterLocal* metrics exhibit large values during non-voiced segments in the case of the original 44.1kHz signal (represented by the green trace). In particular, an *F0semitone* value of 62 represents an F0 of 987Hz, which is deemed implausible. The original 44.1kHz speech signal was resampled to 16kHz using TorchAudio's resample method, which applies sinc-interpolation (Yang et al., 2021).

Our initial hypothesis was that the original 44.1kHZ audio contains high-frequency harmonics (e.g., whirring PC-fan) that are more easily picked-up when OpenSMILE is used in certain configurations (in this case a higher sampling rate). To test this hypothesis, we added high-frequency Gaussian noise of -30dB to the audio to determine if it would reduce the ability

to detect these harmonics. The results for a single segment are depicted in the figure below. The 16kHz resampled data showed an expected outcome; the signal-to-noise ratio at voiced boundaries for the noisy signal, represented by the orange trace of (a), was slightly trimmed at second 16.25 and 16.75, resulting in a decrease of higher jitter values. Conversely, the addition of noise to the 44.1kHz signal, represented by the blue trace of (b), did not result in an improved detection of unvoiced regions. As such, there was no decrease in F0 or jitter values. Hence, we can conclude that resampling high-frequency seems to contribute more to improved voiced boundary detection than the Gaussian-noise addition.

Figure 13

Impact of Gaussian noise superposition to (resampled) audio.

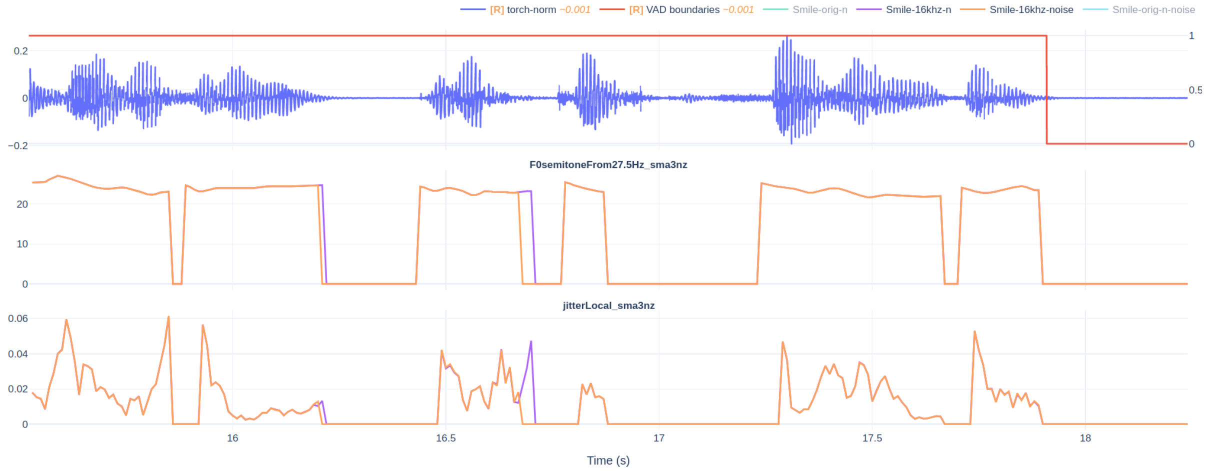

(a) 16kHz

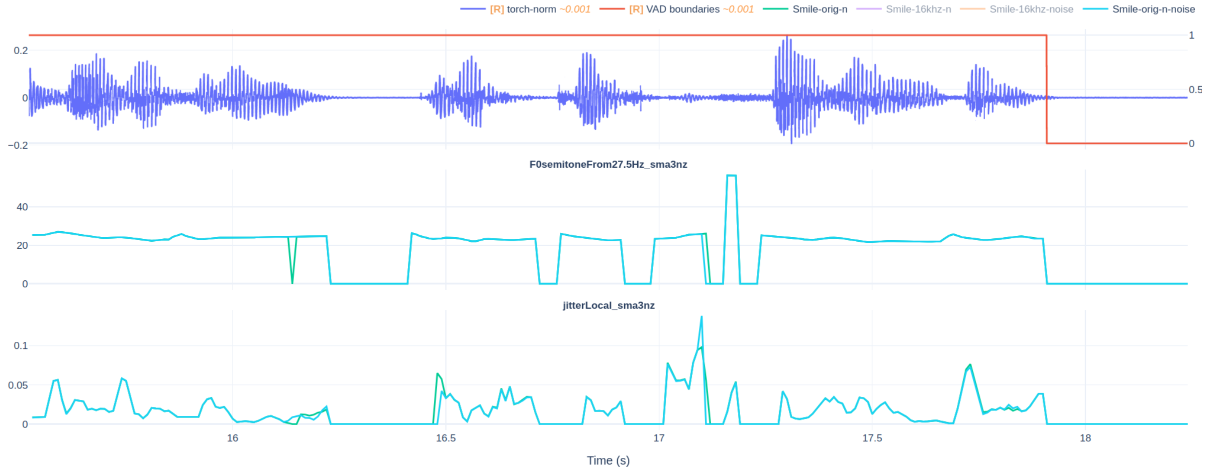

(b) 44.1kHz

S6. OpenSMILE Delta Visualizations

Figure 14

GeMAPSv01b feature subset delta visualizations, divided into temporal (row 1), frequency (row 2), and amplitude (row 3) related features.

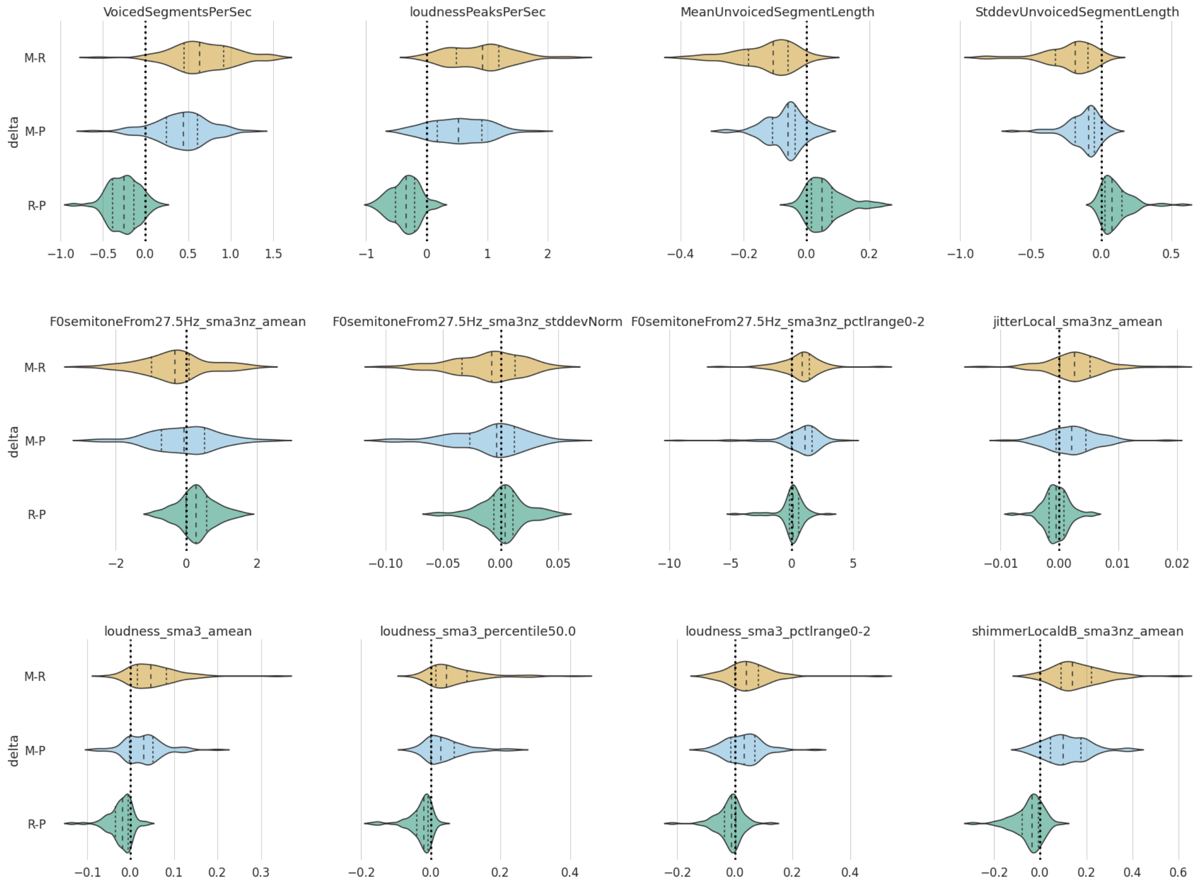

The feature subplots in the above graph exhibit a general trend; the deltas between the unscripted-to-scripted speech styles (i.e., M-R, M-P) have a greater variation and a consistent offset. Conversely, the deltas between the unscripted-to-unscripted tasks (i.e., R-P) have a more limited variation and the offset is closer to the 0 delta value, which suggests a greater similarity in the speech features.

S7. ECAPA-TDNN & GeMAPS Distribution Plots

Figure 15

KDE plot, depicting the distribution of the web application ECAPA-TDDN embeddings. A subset of embedding dimensions was chosen, each displaying a normal distribution.

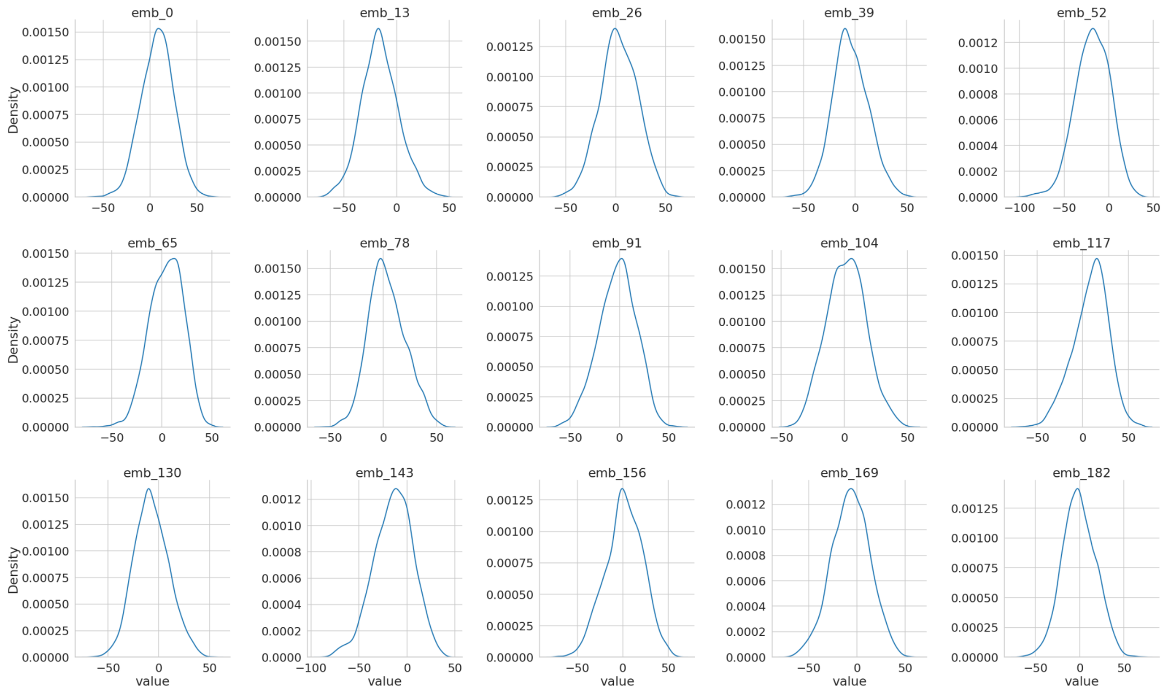

Figure 16

KDE plot of the web application GeMAPSv01b functional features, indicating non-normal distributions.

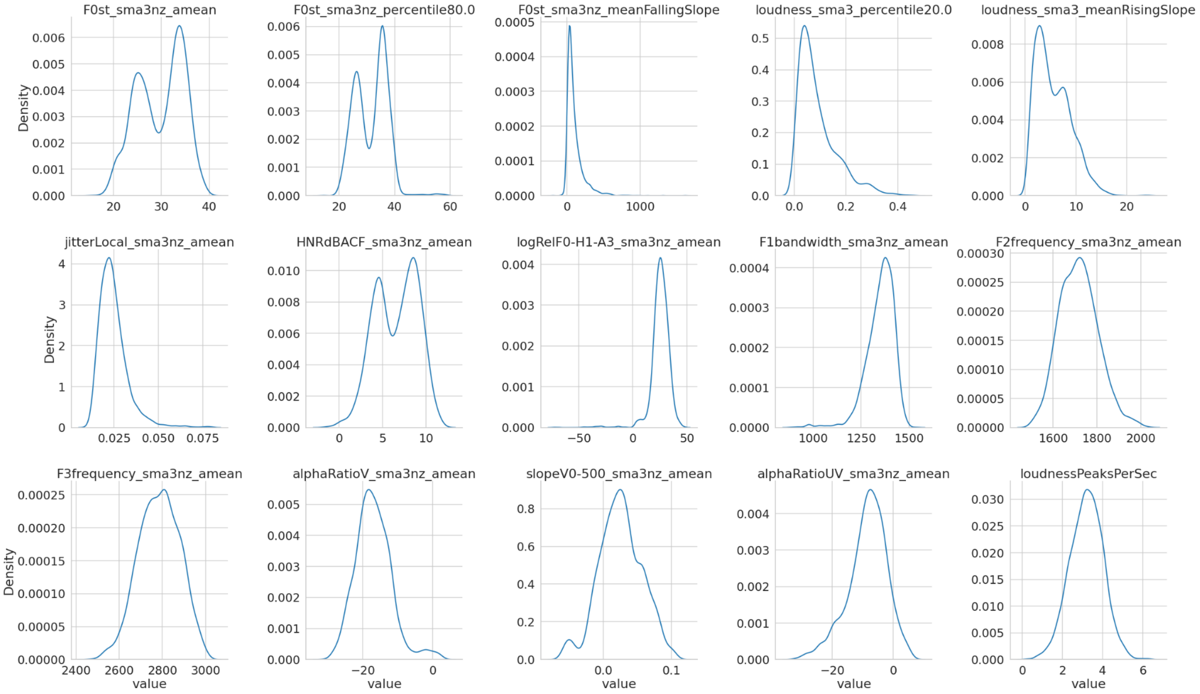

S8. Logistic Regression Weight Coefficients

Figure 17

Visualization of the 15 largest feature coefficients of the logistic regression models that were trained on GeMAPSv01b configuration.

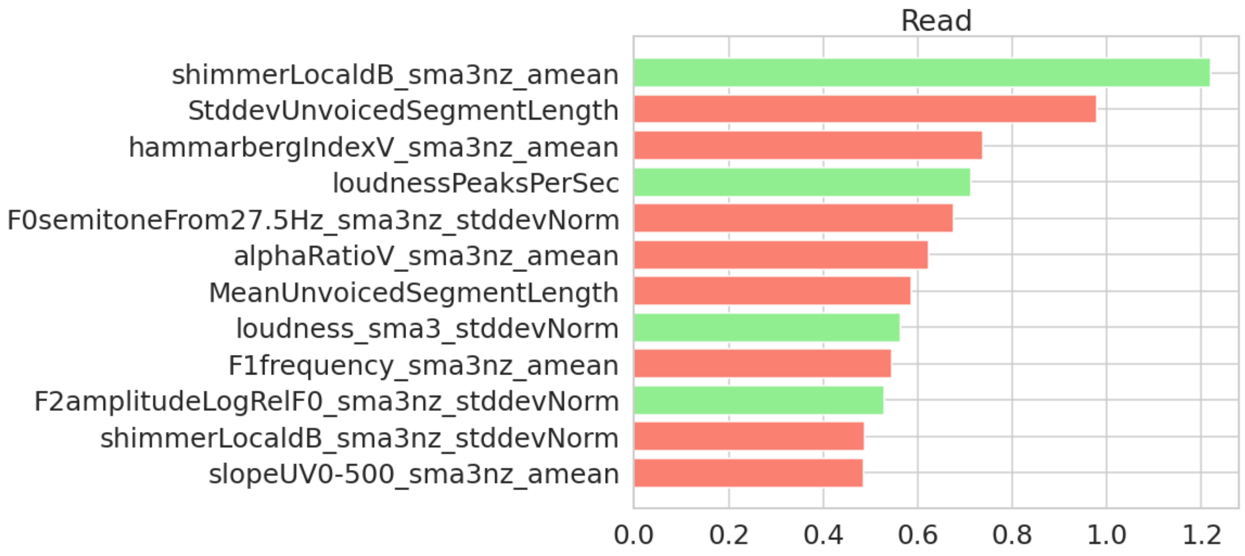

(a) Model trained on the web app data.

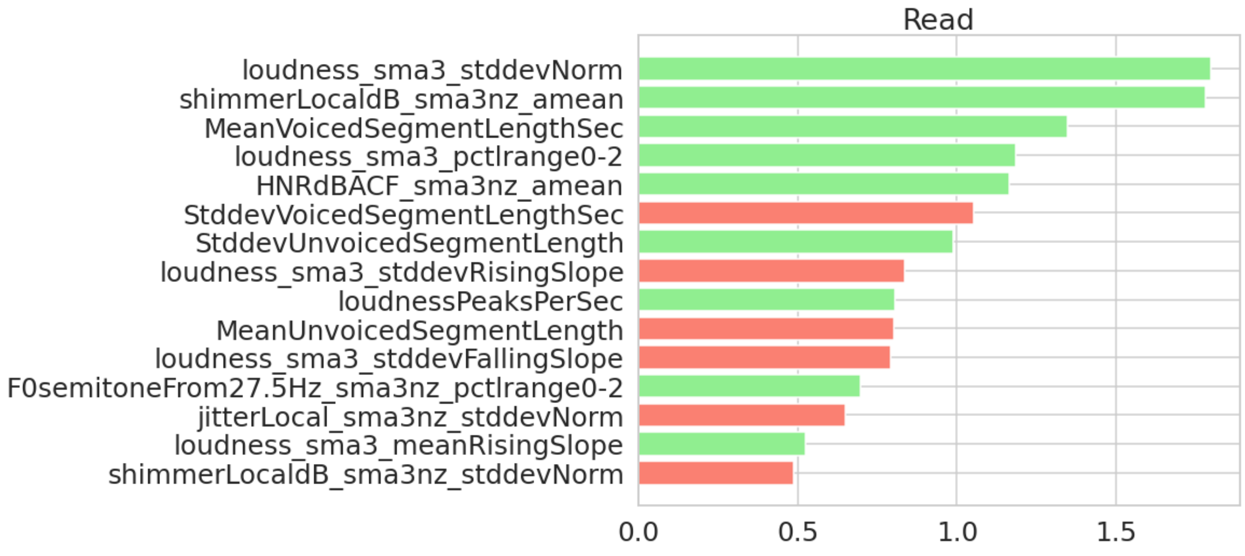

(b) Model trained on CGN data.

Note. The color indicates whether the feature coefficient is positive (green) or negative (red) in relation to the target variable (Read speech).

Remark how the weight coefficient for the "shimmerLocaldB_sma3nz_amean" exhibits a substantial positive value in both subplots. This positive sign for the coefficient can be interpreted as indicating that a decrease in shimmer contributes to a higher likeliness of having read-aloud speech, which contradicts existing literature. It is of particular interest that this trend is also observed when fitting a model on the CGN data (b), suggesting that the OpenSMILE GeMAPSv01b shimmer values tend to decrease as the speech becomes less scripted. The "*jitterLocal_sma3_stddevNorm*" parameter, has a smaller value and is not incorporated in the top 15 values for the CGN model (b).

S9. Effect Size Shimmer & Jitter

S9.1. Shimmer

Figure 18

Effect plot of shimmer (a) and corresponding screenshots of computation process (b-c).

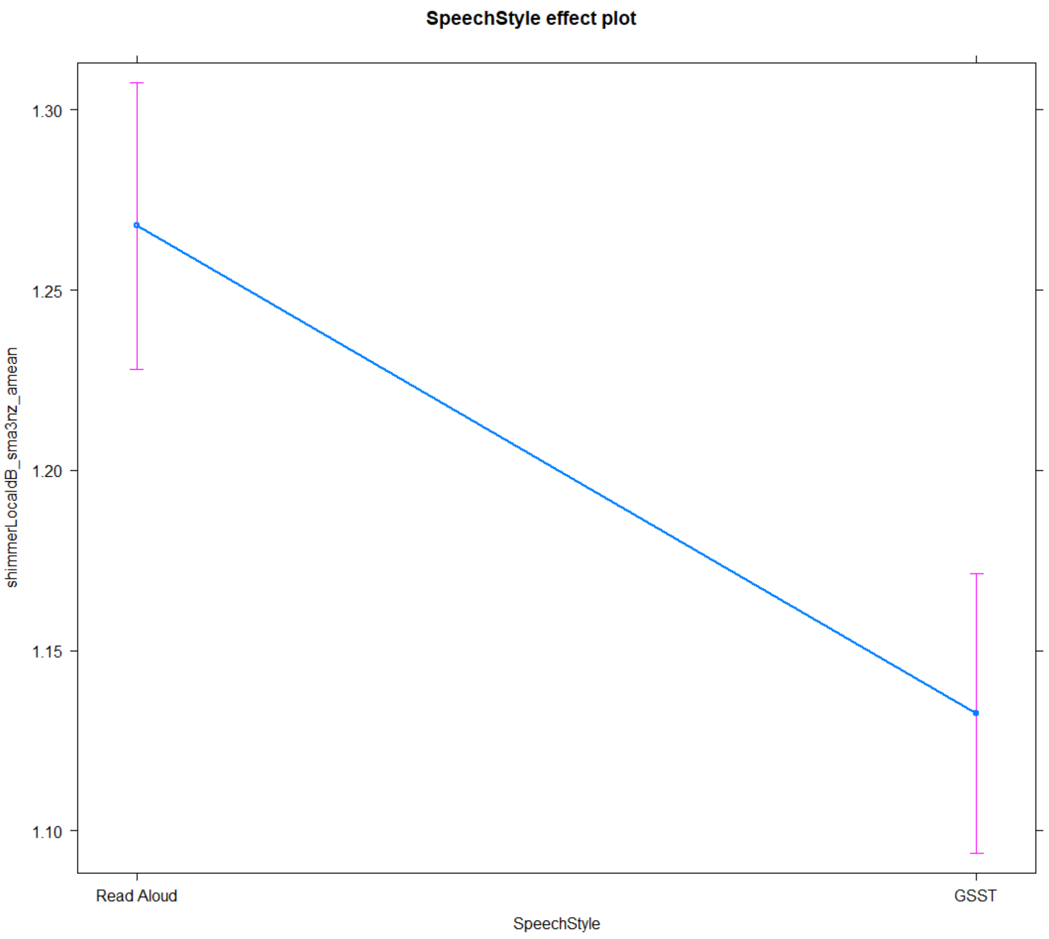

(a) Effect plot of Shimmer.

```
> Anova(d0.1, type = 'III')
Analysis of Deviance Table (Type III Wald chisquare tests)

Response: shimmerLocaldB_sma3nz_amean
             Chisq Df Pr(>Chisq)
(Intercept) 3658.37  1  < 2.2e-16 ***
SpeechStyle  535.32  1  < 2.2e-16 ***
---
Signif. codes:  0 '***' 0.001 '**' 0.01 '*' 0.05 '.' 0.1 ' ' 1
```

(b) ANOVA.

```
> emmeans0.1 <- emmeans(d0.1, pairwise ~ SpeechStyle, adjust ="none", type = "response")
> emm0.1 <- summary(emmeans0.1)$emmeans
> emmeans0.1$contrasts
 contrast           estimate      SE   df t.ratio p.value
 Read Aloud - GSST     0.135 0.00585 2829  23.137  <.0001

Degrees-of-freedom method: kenward-roger
> effSummary <- summary(eff_size(emmeans0.1, sigma=sigma(d0.1), edf=df.residual(d0.1)))
Since 'object' is a list, we are using the contrasts already present.
> effSummary
 contrast            effect.size     SE   df lower.CL upper.CL
 (Read Aloud - GSST)         1.1 0.0496 2829        1      1.2

sigma used for effect sizes: 0.1232
Degrees-of-freedom method: inherited from kenward-roger when re-gridding
Confidence level used: 0.95
```

(c) Effect size summary.

S9.2. Jitter

Figure 19

Effect plot of jitter (a) and corresponding screenshots of computation process (b-c)

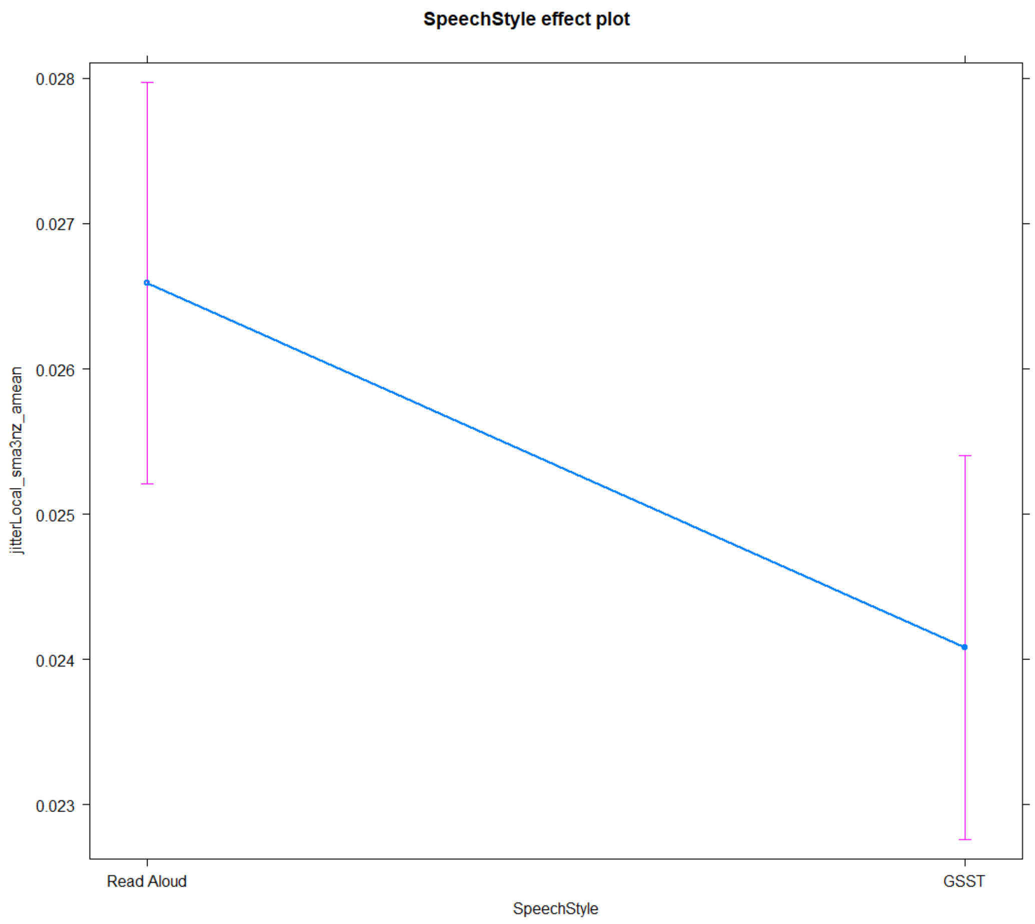

(a) Effect plot of jitter.

```
> Anova(d0.1, type = 'III')
Analysis of Deviance Table (Type III Wald chisquare tests)

Response: jitterLocal_sma3nz_amean
               Chisq Df Pr(>Chisq)
(Intercept) 1399.394  1  < 2.2e-16 ***
SpeechStyle   88.301  1  < 2.2e-16 ***
---
Signif. codes:  0 ‘***’ 0.001 ‘**’ 0.01 ‘*’ 0.05 ‘.’ 0.1 ‘ ’ 1
```

(b) ANOVA.

```
> emmeans0.1 <- emmeans(d0.1, pairwise ~ SpeechStyle, adjust ="none", type = "response")
> emm0.1 <- summary(emmeans0.1)$emmeans
> emmeans0.1$contrasts
 contrast           estimate       SE   df t.ratio p.value
 Read Aloud - GSST  0.00251 0.000267 2831   9.397  <.0001

Degrees-of-freedom method: kenward-roger
> effSummary <- summary(eff_size(emmeans0.1, sigma=sigma(d0.1), edf=df.residual(d0.1)))
Since 'object' is a list, we are using the contrasts already present.
> effSummary
 contrast             effect.size     SE   df lower.CL upper.CL
 (Read Aloud - GSST)        0.446 0.0478 2831    0.352     0.54

sigma used for effect sizes: 0.005624
Degrees-of-freedom method: inherited from kenward-roger when re-gridding
Confidence level used: 0.95
```

(c) Effect size summary.

S10 Image stimuli Analysis

Figure 20

OpenSmile feature subset violin plot per image stimuli

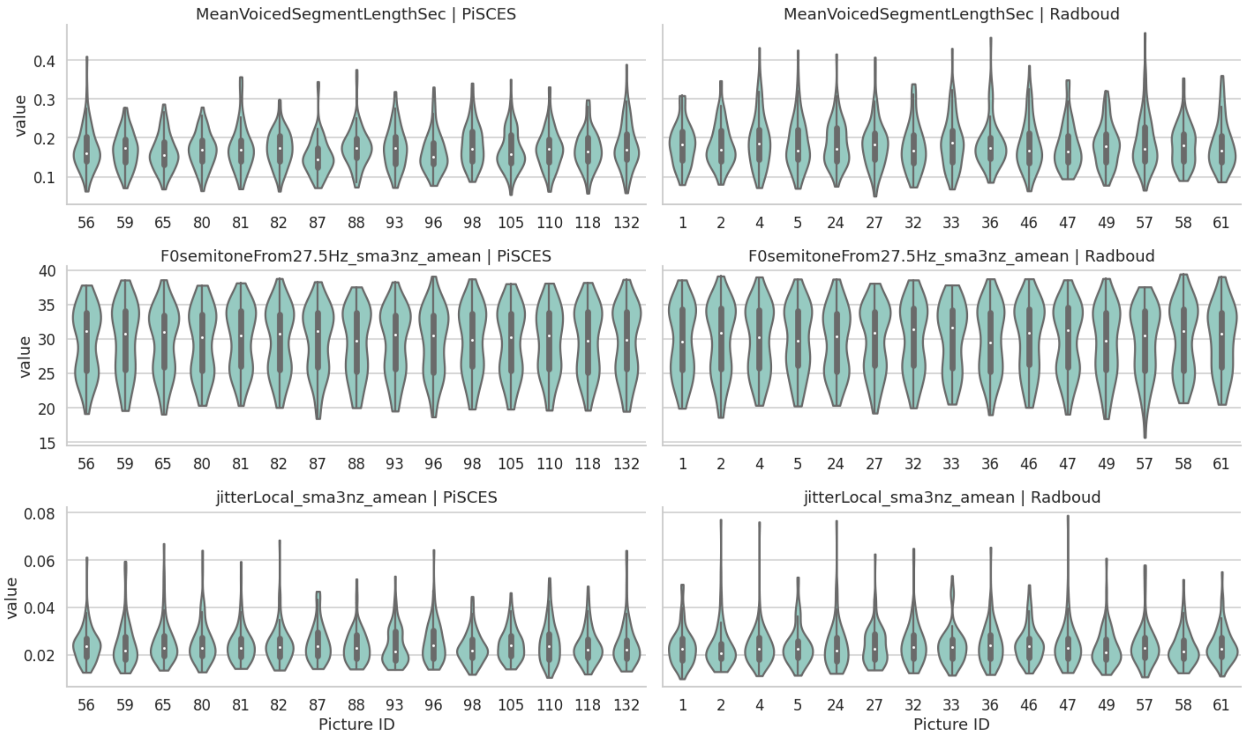

S11. Session Number Analysis

Figure 21

Illustration of voiced duration, start offset (time from pressing 'start' to the first voiced segment), and end offset (time from last voiced segment to pressing 'stop')

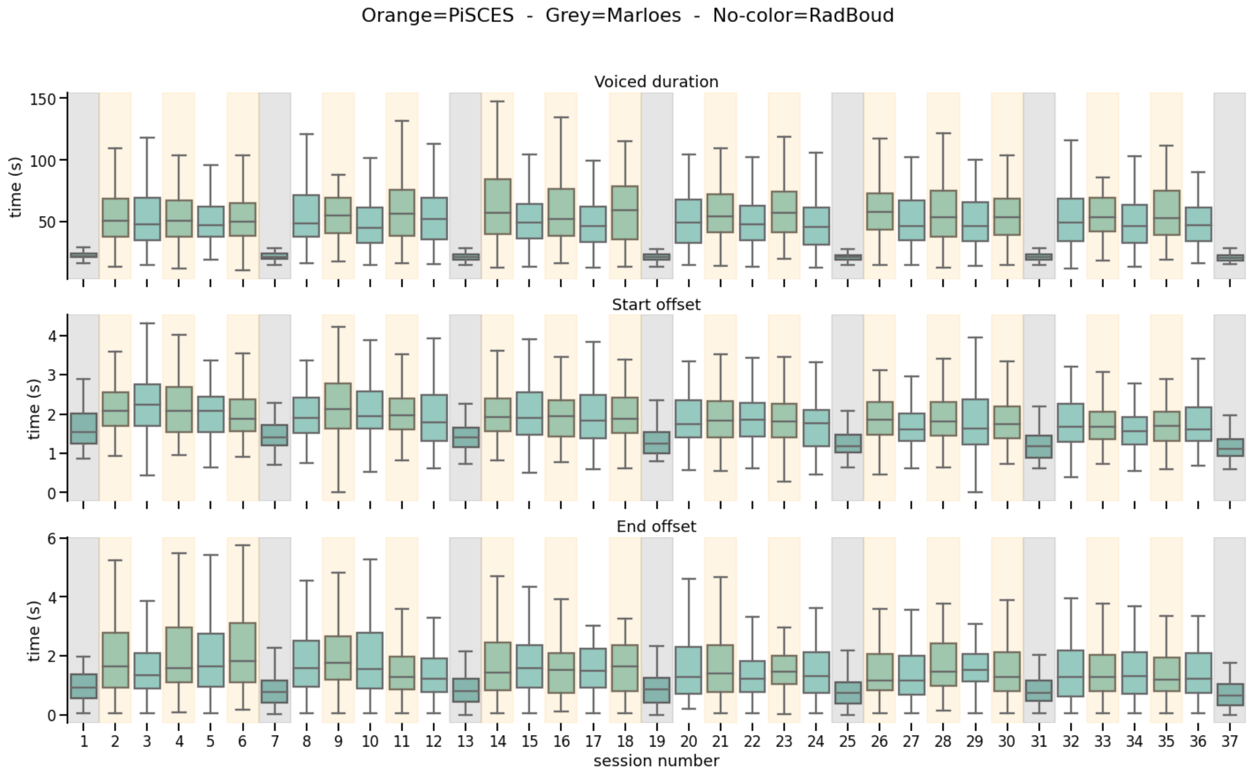

(a) Relative to task number.

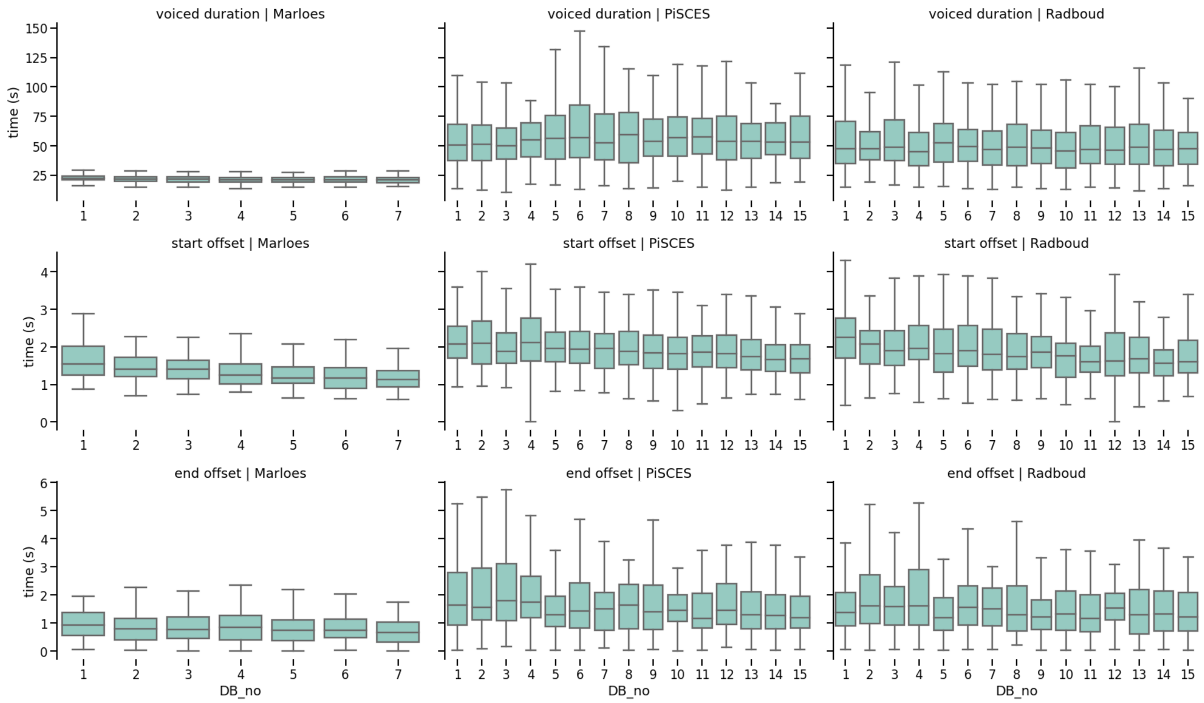

(b) *Relative to Database Number (DB_no), i.e., the occurrence number within each stimulus-type (Radboud, PiSCES, Marloes)*
